# Nutrimetabolomics reveals food-specific compounds in urine of adults consuming a DASH-style diet

**DOI:** 10.1038/s41598-020-57979-8

**Published:** 2020-01-24

**Authors:** Nichole A. Reisdorph, Audrey E. Hendricks, Minghua Tang, Katrina A. Doenges, Richard M. Reisdorph, Brian C. Tooker, Kevin Quinn, Sarah J. Borengasser, Yasmeen Nkrumah-Elie, Daniel N. Frank, Wayne W. Campbell, Nancy F. Krebs

**Affiliations:** 1 Department of Pharmaceutical Sciences, School of Pharmacy and Pharmaceutical Sciences, University of Colorado Anschutz Medical Campus, 12850 East Montview Boulevard, Aurora, CO 80045 USA; 2 Mathematical and Statistical Sciences, University of Colorado Denver, Denver, Colorado, USA; 3 Department of Pediatrics, Section of Nutrition, University of Colorado Anschutz Medical Campus, 12700 E 19th Avenue, Aurora, CO 80045 USA; 4 Department of Medicine, Division of Infectious Diseases, University of Colorado Anschutz Medical Campus, 12700 E. 19th Ave, Aurora, CO 80045 USA; 5 Department of Nutrition Science, Purdue University, 700 West State Street, West Lafayette, IN 47907 USA

**Keywords:** Diagnostic markers, Metabolomics

## Abstract

Although health benefits of the Dietary Approaches to Stop Hypertension (DASH) diet are established, it is not understood which food compounds result in these benefits. We used metabolomics to identify unique compounds from individual foods of a DASH-style diet and determined if these Food-Specific Compounds (FSC) are detectable in urine from participants in a DASH-style dietary study. We also examined relationships between urinary compounds and blood pressure (BP). Nineteen subjects were randomized into 6-week controlled DASH-style diet interventions. Mass spectrometry-based metabolomics was performed on 24-hour urine samples collected before and after each intervention and on 12 representative DASH-style foods. Between 66–969 compounds were catalogued as FSC; for example, 4-hydroxydiphenylamine was found to be unique to apple. Overall, 13–190 of these FSC were detected in urine, demonstrating that these unmetabolized food compounds can be discovered in urine using metabolomics. Although linear mixed effects models showed no FSC from the 12 profiled foods were significantly associated with BP, other endogenous and food-related compounds were associated with BP (N = 16) and changes in BP over time (N = 6). Overall, this proof of principle study demonstrates that metabolomics can be used to catalog FSC, which can be detected in participant urine following a dietary intervention.

## Introduction

Human nutrition research includes controlled-feeding strategies to evaluate associations between consumption of specific foods or diets and health indicators. Recent advances in metabolomics make it possible to gather data on a multitude of foods and biosamples^1–4^. Nutrimetabolomics, which represents the intersection of metabolomics and nutrition research, offers an opportunity to investigate the effects of whole diets, specific foods, and food components on the human metabolome^5^. For example, Rebholz, *et al*. applied metabolomics to identify serum markers of participant adherence to consuming a Dietary Approaches to Stop Hypertension (DASH) diet^3^. A novel aspect of the Rebholz, *et al*. study was their effort to define a panel of markers indicative of a DASH-style eating pattern. Similarly, Gordon-Dseagu, *et al*. used metabolomics to explore the relationship between plasma markers, sleep, and a DASH-style diet^6^. These, and other studies^2,7,8^, support the proof-of-principle that metabolomics can discover and link biomarkers of food intake, from both whole diets and individual foods, to health outcomes.

Controlled-feeding studies are essential for understanding how diets, individual foods, and food constituents are related to indices of human health. However, the complexity of diets, limited understanding of chemical compositions of foods, shortage of food-specific biomarkers, and personalized nature of human metabolism limit the generalizability of results. Despite these challenges, nutrimetabolomics holds promise for discovering and validating food-derived biomarkers that can be assigned, for example, as biomarkers of intake and/or biomarkers of effect; either of which may associate with health indicators^5^. The process of discovering and validating specific biomarkers of food intake is extensive, usually entailing well-controlled, acute feeding of a specific food^9^. An alternative is to first identify what compound(s) designate a food as unique by comparing the chemical composition of various foods using mass spectrometry-based metabolomics. The patterns of these “food-specific compounds” (FSC) can then be traced in human biospecimens following whole diet interventions, again using metabolomics. FSC that associate with ingestion of specific foods from the whole diet can be classified as candidate biomarkers of food intake, potentially eliminating the need for acute feeding of a single food. While various terminologies are still being explored in the field of nutrimetabolomics^10^, we are defining FSC as compounds detected in only one food and not in any other food present within the study diet. This alternative strategy, which in summary entails the discovery of FSC first in foods before tracing them back to biospecimens, is made possible by the comprehensive and high-throughput nature of metabolomics. Further, FSC can be associated with health indices to discover biomarkers of effect.

The purpose of this project was to provide proof-of-principle that a metabolomics-based strategy for biomarker discovery is plausible, by verifying that FSC can be found in urine following consumption of certain foods. We leveraged existing data and samples from a completed, randomized, controlled-feeding study originally designed to assess the effects of consuming a DASH-style diet containing different predominant sources of protein on changes in blood pressure (BP)^11^.

The present study sought to: (1) characterize the chemical composition of selected foods consumed by participants in a DASH-style diet study using LC/MS; (2) determine if any of the compounds detected in these foods were unique to an individual food (i.e. categorize compounds as FSC); and (3) establish if FSC could be detected in 24-hour urine samples obtained from the study participants. To our knowledge, this study is unique in several ways. It is one of the first to provide a comprehensive, metabolomics-based characterization of compounds in multiple foods that were actually consumed by participants in a nutritional intervention study. Second, it is novel in using metabolomics to identify and trace compounds that are unique to foods back to human specimens. Finally, it is possibly the first to generate “food-specific signatures”, which comprise a sum of the FSC. Although not novel, we also performed association analysis between urinary compounds and BP levels. We hypothesized that FSC and/or other, food-related or endogenous compounds (i.e. non-FSC) in urine would be associated with BP and changes in BP after consuming the DASH-style diet.

## Experimental Section

The present study was carried out in accordance with the ethical principles of the Declaration of Helsinki/relevant guidelines and regulations. Extensive detail is included in the Supplemental Methods.

### Subjects and experimental design

Nineteen individuals (6 men and 13 women), recruited from the greater Lafayette, IN, region, provided informed consent to participate in a randomized, crossover, controlled feeding study, and completed the study protocol [mean ± SEM age: 61 ± 2 years; body mass index 31.2 ± 1.4 kg/m^2^]^11^. The protocol was approved by the Purdue University Biomedical Institutional Review Board and was registered at clinicaltrials.gov: NCT01696097 (09/26/2012). During the 18-week study period, participants initially consumed their self-chosen, unrestricted (typical) diets for two weeks. Participants were then randomly assigned to consume a DASH-style diet with either lean pork or chicken and fish as the predominant sources of dietary protein for six weeks. This time period was followed by a four-week washout period during which participants again consumed their typical diets prior to consuming the DASH-style diet containing the other predominant protein sources (Supplemental Fig. S1)^11^. Samples utilized for the current research consisted of 12 individual foods consumed during the DASH-style diet and urine samples collected prior to each intervention and during the final two weeks of each controlled feeding period.

### Blood pressure measurement

Twenty-four-hour ambulatory systolic BP (SBP) and diastolic BP (DBP) were measured for 3 consecutive days during each of the four measurement periods using a 24-h ambulatory BP monitoring system (SunTech Medical, Inc., Morrisville, NC, USA). Blood pressure measurements were made at 30-min intervals during the day (0800–2100) and at 90-min intervals during the night (2100–0800). A female subject was missing BP measurements for the second controlled feeding period resulting in no data for this participant for this period.

### Urine collection

Twenty-four-hour urine collections were obtained before and near the end of each of the two DASH-style diet periods. A total of 76 urine samples were used for untargeted metabolomics analysis, including 38 collected while the 19 participants consumed their typical diets (19 before each intervention period) and 38 while the DASH-style diet was consumed (19 during each intervention period). For simplicity, these are referred to as pre- and post-diet urine samples.

### Food preparation

The DASH-style diets consisted of seven daily menus of specified foods and beverages for each of the two 6-week controlled feeding periods (Supplemental File- DASH Macros and Menu). Participants consumed the prescribed menus on a 7-day rotating cycle during each controlled feeding period. Twelve fresh, whole foods were selected from the menus and portions stored at −80 °C until analysis. Because the original study assessed the effects of different protein sources consumed with a DASH-style diet on BP responses, all four types of meat included in the diet (pork loin, chicken, fish, and beef loin) were analyzed. Fruits and vegetables, if not pre-washed, were washed with tap water and prepared with inedible parts (i.e. leaves or peels) removed. All foods were purchased at a grocery store in West Lafayette, IN, prepared for storage in a metabolic research kitchen at Purdue University, stored in a −80 °C freezer and shipped with dry ice to the University of Colorado Anschutz Medical Campus, where lyophilization and methanol extraction were performed.

### Sample preparation

Chemicals, standards, and reagents are described in Supplemental Methods. Detailed urine and food sample preparation methods are presented in Supplemental Methods and Table S1. Briefly, approximately 50 mg of each freeze-dried food sample was prepared for metabolomics using methods similar to those published previously^12^. In the case of coffee, 10 µL of coffee was diluted with 90 µL of water prior to analysis. To each sample, 480 μL of chilled methanol and 10 µL of labeled or non-endogenous compound standards were added and proteins allowed to precipitate at −80 °C for 60 minutes. Following centrifugation, supernatants were transferred to new microcentrifuge tubes and dried using vacuum centrifugation. Each sample was suspended in 50 µL of 95:5 LC/MS grade water-acetonitrile and samples stored at −80 °C until analysis.

### Liquid chromatography mass spectrometry

Urine and individual foods were analyzed by reverse phase LC/MS as described^12^. Briefly, 1 µL of a food or urine sample was injected onto a C18 column attached to an Agilent 1290 series high performance liquid chromatography (HPLC) pump, except for peanut butter which used a volume of 8 µL. MS was performed using an Agilent 6520 Time-of-Flight-MS with dual electrospray ionization source (Agilent Technologies, Santa Clara, CA, USA). Quality control was performed as described in Supplemental Methods.

### Data processing

Untargeted data mining was performed using MassHunter Profinder (Agilent Technologies)^12,13^. Data were imported into Mass Profiler Professional (MPP, Agilent Technologies) for processing and analysis. Prior to analysis, compounds present in preparation blanks and instrument blanks were removed from the dataset. Because there was high variability in the food matrices, no normalization was used when foods and urines were analyzed together. However, for the analysis of urine alone, data were normalized using total useful signal^14^. Following extraction and processing, the resulting numerical values for compounds have no units and are expressed as “compound abundances” throughout the manuscript. Principle Components Analysis (PCA) was conducted using filtered data prior to statistical analysis. Hierarchical clustering using Ward’s method and Venn diagrams were generated using filtered and non-normalized data in MPP.

### Compound annotation

The tool, ID Browser within MPP was used to putatively annotate compounds with common chemical names using isotope ratios and an error window of <10 ppm. This software utilizes an in-house database comprising data from METLIN, Human Metabolome Database (HMDB), Kyoto Encyclopedia of Genes and Genomes (KEGG), Lipid Maps, and data from our authentic standards database, to match masses from unnamed compounds to database entries. Food compounds were also searched against the food database, FooDB. A full list of food compounds is included as Supplemental File “Uncurated Food Compound Lists”. Compounds that matched to a database entry represent a Metabolomics Standards Initiative level 3 identification, based on the proposed minimum reporting by Sumner, *et al*.^15^ and hence compound names are considered putative (i.e. tentative). Urinary FSC, associated with BP were subjected to tandem MS to improve confidence in the database matching based on fragmentation information. Resulting MS/MS fragment spectra were searched against our in-house standards database, METLIN and NIST14 MS/MS spectral libraries^16^. Unidentified MS/MS data were exported to SIRIUS 4.0.1 and the spectral data searched using the theoretical compound fragment patterns in CSI:FingerID^17^. Compounds matched to spectral libraries and theoretical fragmentation databases represent a Metabolomics Standards Initiative level 2–3 identification^15^.

### Classification of FSC

MPP was used to determine which compounds could be classified as FSC by comparing the intersection of compounds present in a single foods vs. the other 11 foods in the study. For the purpose of this study, compounds were designated as FSC if they were unique to a food within this dataset of 12 study foods. Results are compiled in Supplemental File- FSC.

### Statistical methods

Statistical analyses were completed in R Studio using R v.3.5.1. Linear mixed effects models were run using the function lme from the nlme package version 3.1–131.

### Relative metabolism of individual foods

For the seven foods consumed consistently in both DASH-style diet periods (apple juice, beef tenderloin, blueberries, broccoli, cucumber, grapefruit, and peanut butter), we summed the abundances of FSC that were also found in urine (Table 1 column 5) to derive an index for each of the 12 individual foods; this index is termed “food-specific signature” for simplicity. In the current study, individual foods were consumed on the same day each week by participants; however, the days of urine collection varied by participant. Further, most FSC were observed in only a few individuals. Summing FSC enabled us to determine signature levels based on the time since consumption (More detail is provided in Supplemental Methods). For each of the seven foods, a linear mixed effects model was used with individual participant as the random effect, the food-specific signature as the outcome, and number of days between when a specific food was consumed and when the urine collection occurred as the predictor. Significance was assessed at a Bonferroni correction for the seven foods (α = 0.05/7 = 0.00714). Models also were run removing outliers and adjusting for batch as a sensitivity analysis to ensure consistency.

**Table 1:** Numerical summary of compounds detected in foods and urine.

1. Food	2. Total # of compounds detected in food	3. # of FSC in food (# of annotated)	4. # of FSC found in urine
Apples	754	108 (47)	20
Apple Juice	702	90 (40)	18
Apples or Apple Juice	1146	254	47
Beef	1003	204 (75)	32
Blueberries	1334	344 (178)	64
Broccoli	1622	468 (181)	82
Chicken	914	219 (61)	16
Coffee	682	209 (93)	74
Cucumber	1645	421 (188)	90
Grapefruit	2292	969 (425)	190
Peanut Butter	1989	922 (414)	164
Pork	730	66 (28)	14
Tilapia	531	74 (25)	13

Following LC/MS and data processing, Mass Profiler Professional (MPP) was used to analyze and summarize data from individual foods. The total number of compounds detected in each food is listed in column 2. MPP was used to compare compounds found in each individual food to the remaining foods to generate a list of compounds that were unique to that food within in the dataset (i.e. FSC, Column 3). The number of FSC annotated using database searches is indicated in parantheses. The database annotations for FSC were manually reviewed using a variety of webtools to determine if they had either been previously detected in that food or were likely to be in that food (Supplemental File- FSC). Finally, MPP was used to determine what FSC were also detectable in study particiant’s urine (Column 4). A full list of the FSC is included as Supplemental File- FSC.

### Association of urinary compounds with blood pressure

To confirm the previously reported effects of consuming the DASH-style diet on SBP and DBP^11^, we used a linear mixed effects model with a random intercept for individual participant, BP level post-diet as the outcome, and pre-diet BP, predominant dietary protein consumed with the DASH-style diet, order of predominant dietary protein consumed, and period of intervention as predictors.

We used two approaches to determine if associations exist between urinary compounds and BP levels in these participants as detailed in Supplemental Methods. The first approach assessed if the relative abundance of each FSC or other compound in urine was associated with SBP or DBP. Data from 74 measurements (18 subjects, each with four measurement periods and 1 subject with two measurement periods) were included. We used a linear mixed effects model with a random intercept for individual participants and BP level as the outcome. The second approach assessed if the relative abundance of each FSC or other compound detected in urine while the participants consumed the DASH-style diet was associated with changes over time in SBP or DBP during the controlled feeding periods (post-diet BP minus pre-diet BP). Data from 37 urine samples and 37 BP change values were included. We used a linear mixed effects model with a random intercept for individual participant, change in SBP or DBP as the outcome, and compound abundance in urine collected while the participants consumed the DASH-style diets and batch as predictors.

## Results

### Summary of food and urinary compound detection

**Figure 1:**
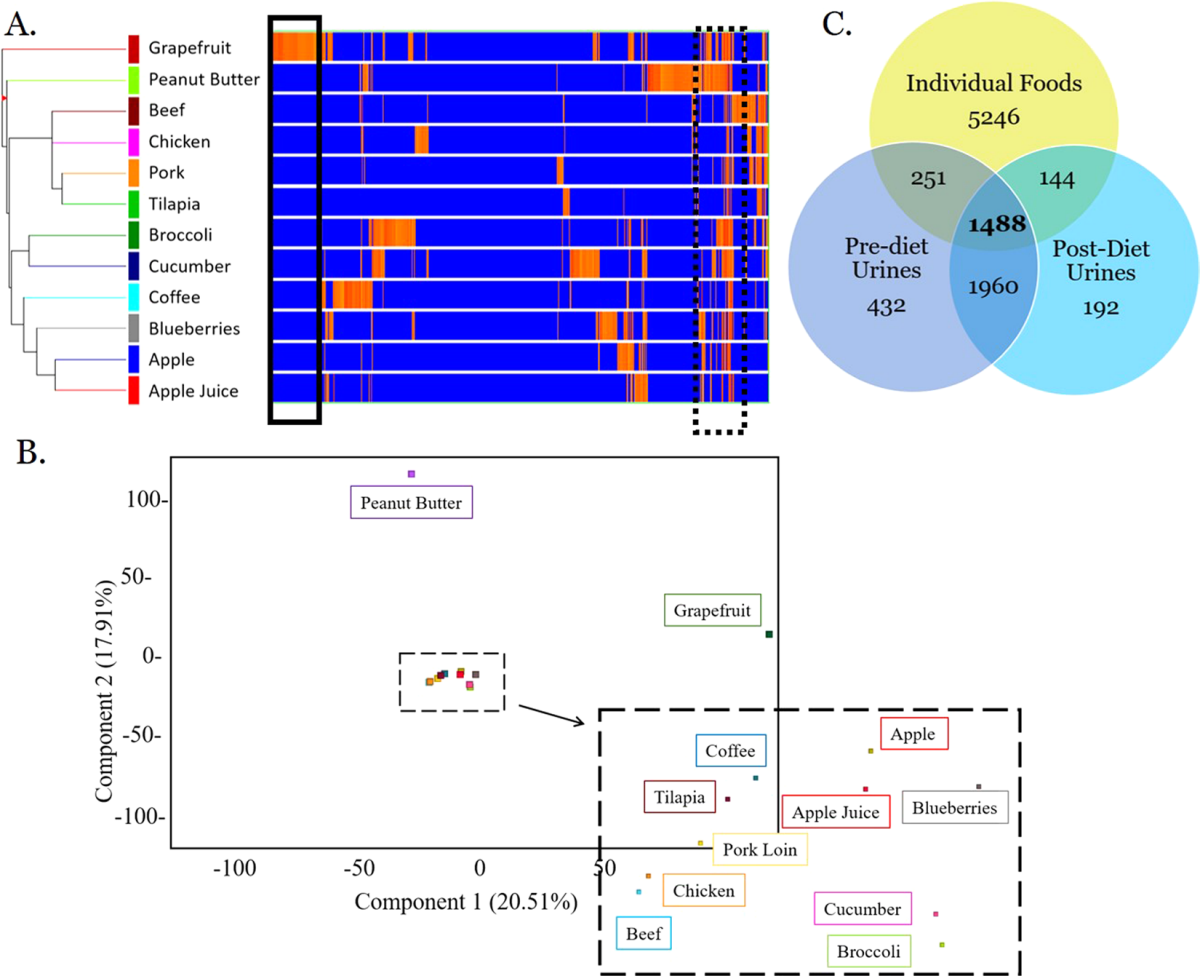
Relationship between individual foods and urine samples visualized using hierarchical clustering (**A**), PCA (**B**), and Venn (**C**). Following metabolomics analysis, a variety of visualization techniques were applied to the dataset. (**A**) Hierarchical clustering of data from all 12 individual foods. The x-axis corresponds to individual compounds detected in the foods which are listed on the y-axis. Blue lines indicate less relative abundance for that compound compared to all other foods while orange/red lines indicate higher relative abundance for that compound compared to all other foods. The vertical distance between where foods split is a rough estimation of their similarity. Solid black box indicates a region of compounds that appear to be unique to grapefruit. Dotted black box highlights a region of compounds that appear to be in common among many foods. (**B**) PCA was performed using data from all foods. Component 1, which explains 20.51% of the variation, is shown on the x-axis and component 2, which explains 17.91% of the variation, is shown on the y-axis. The first 4 PCs explain approximately 63% of the variation. (**C**) Venn diagram illustrates overlap between the 7,089 compounds detected in individual foods (green circle), the 4,091 compounds detected in pre-diet urine (grey-blue), and the 3,744 compounds detected in post-diet urine. A total of 1,488 compounds were detected in all 3 sample types. A total of 1960 compounds were detected in both pre- and post-diet urine.

Following subtraction of potential contaminants or artifacts, a total of 4,467 compounds were detected among 76 urine samples and a total of 7,089 compounds were detected in 12 individual foods. Hierarchical clustering of compounds from individual foods showed distinct differences in their compound composition (Fig. 1A). For example, grapefruit appears to have many unique compounds compared to other foods in the study (Fig. 1A, solid black inlaid box). Conversely, there are groups of compounds that are shared among many or all foods (Fig. 1A, dotted black box). The distance between foods is a rough estimation of their similarity, as visualized by the nodes and branches on the left of Fig. 1A. For example, apple and apple juice cluster close together as do the 4 types of meat (beef, chicken, pork, and tilapia), and the two vegetables (cucumber and broccoli). As shown in the expanded black dotted box and Fig. 1B, the four types of meat cluster away from plant products. Examination of the PCA loadings plot indicates distinct clusters of compounds that co-occur in multiple foods (Supplemental Fig. S2).

When metabolomics data from pre- and post-diet urine samples were analyzed, 1,960 compounds were detected in both pre-diet and post-diet urine (Fig. 1C). Overall, 432 compounds were found only in the uncontrolled, pre-diet urines compared to 192 that were found only in the post-diet urines. A total of 1,488 compounds were observed in both individual foods and urine (Fig. 1C), while 5,246 compounds were detected in food but not urine. A significant overlap (>90%) of compounds was found between the pork and chicken/fish composite diets (data not shown).

### Summary of FSC

Analysis of individual foods showed a range of 531 compounds detected in tilapia to 2,292 compounds detected in grapefruit (Table 1, column 2). Depending on the food, between 66–969 compounds were classified as FSC (Table 1, column 3). For example, of the 754 compounds detected in apple, 108 of these were unique to apple when compared to the remaining 11 foods. A complete list of FSC is provided in Supplemental File- FSC. A range of 5–66 FSC had been previously reported in the food of interest (Supplemental File- FSC). For example, 20 of the 468 FSC detected in broccoli were previously reported in broccoli or the *Cruciferous/ Brassicaceae* family, according to web-based tools such as HMDB and FooDB.

Close inspection of the data revealed some notable patterns. For example, 5 of the FSC detected in peanut butter were putatively identified as being exogenous; for example, these were labeled as additives, emulsifiers, flavorings, antibiotics, or pesticides (Supplementary File- FSC). Conversely, no FSC from blueberries, broccoli, grapefruit, or pork appeared to be exogenous. As expected, lipid molecules were predominantly detected in the 4 meats and peanut butter while the fruits and vegetables contained few lipids but a high number of phenolic compounds (Supplementary File- FSC).

### Food compounds and FSC in urine

We next searched for the presence of FSC in pre- and post- diet urine samples. When FSC found in at least one of the 37 post-diet urine samples were considered, anywhere from 13 (tilapia) to 190 (grapefruit) FSC were also found in urine (Table 1, column 4), representing unmetabolized food compounds from these foods.

To further clarify the overlap between food-derived compounds and urine, the analysis was expanded to include all compounds that had been detected in any of the 12 foods. When compounds present in at least one urine sample were considered, the number of food compounds detected in pre- vs post- diet urine samples did not differ notably (Supplemental Table 2, column 2 vs 3). For example, there were 4,091 food-derived compounds present in at least one of the pre-diet urine samples and 3,744 compounds present in at least one of the post-diet urine samples. When considering inter-individual similarity, 280 compounds were found in at least 10 of the 19 participants’ post-diet urine samples (50%); between 48–95 (17–34%) of these compounds were also detected in at least one food. Finally, 44 compounds were observed in the majority of the 19 participants’ urine samples (80%); between 4–17 (9–39%) of these compounds were also found in at least one food. There was an increase in compounds from individual foods detected in the post-diet urines compared to the pre-diet urines, when compounds found in >80% of urine samples are considered. For example, 17 broccoli compounds (FSC and non-FSC) were detected in at least 80% of the pre-diet urines and 20 broccoli compounds were detected in at least 80% of the post-diet urines.

### Relative metabolism of individual foods using metabolomics data

**Figure 2:**
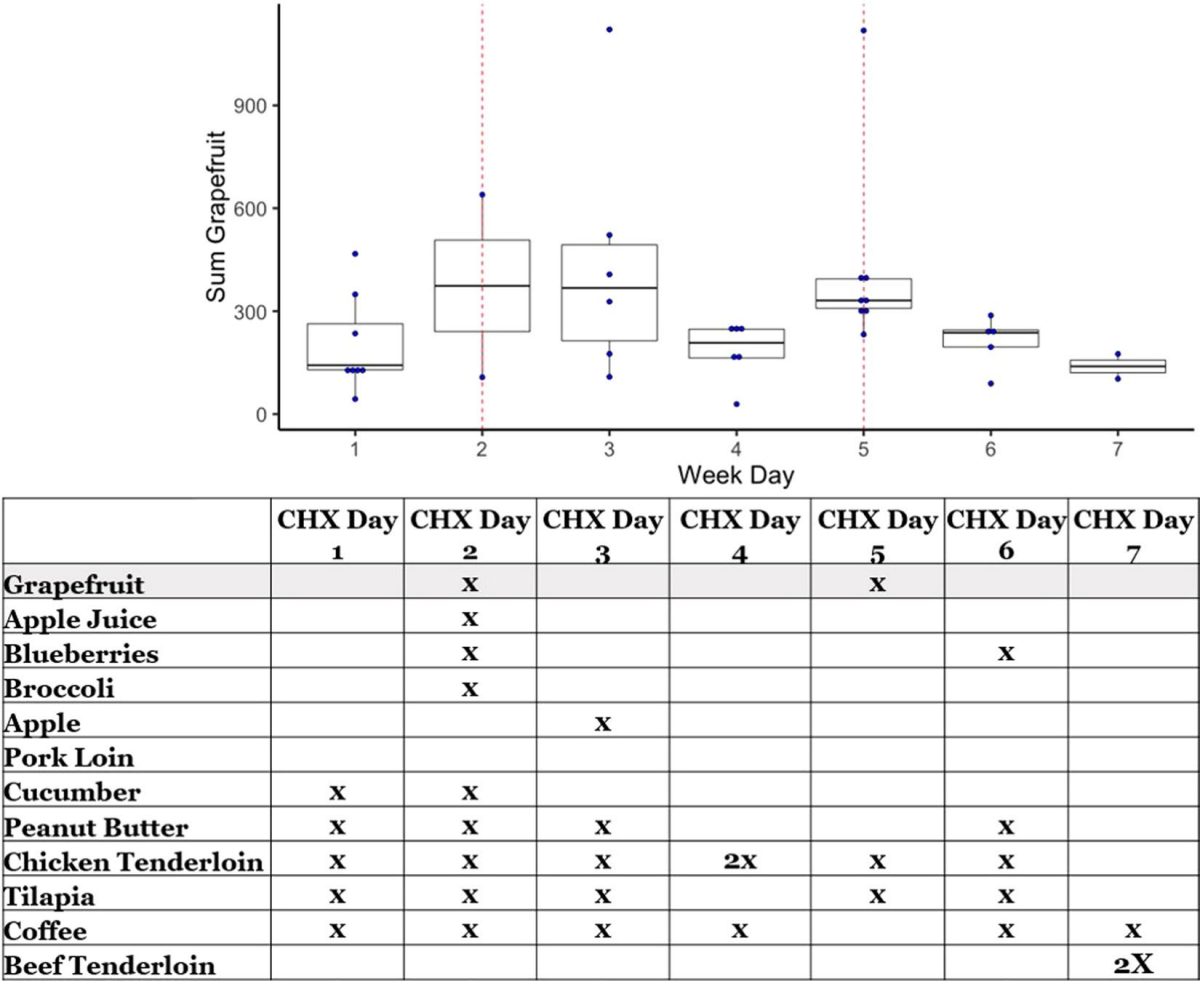
Relative metabolism of food-specific compounds (FSC) detected in urine. Following analysis of food samples using LC/MS, data were analyzed to determine what compounds were FSC. The aggregate of FSC for each food was considered a food-specific-signature. The abundance values for the FSC that comprised a signature were summed and used to determine relative metabolism for each food. The graph shows the intensity of the grapefruit signature plotted over time. The table illustrates the day each food was consumed, with grapefruit, for example, being consumed on days 2 and 5. (CHX = Chicken).

Data were also used to determine if urinary levels of FSC were related to the time a food was ingested. For this purpose, urinary FSC (Table 1, column 4) were used to determine if a linear relationship existed between a food-specific signature and time since consumption. A “food-specific signature” is defined here as an aggregate of all FSC for a food that were also detected in urine and includes the total sum of that food’s FSC abundance values in each individual. Aggregation enabled inclusion and analysis of rare FSC (i.e. those seen in only a few individuals). For example, plotting the intensity of the “grapefruit signature”, which was consumed on days 2 and 5 of both chicken/fish- and pork-based interventions, over time showed higher levels of grapefruit urinary FSC on the days of consumption **(**Fig. 2**)**, with degradation of this signature over time. A Bonferonni adjusted significant linear relationship was found for the grapefruit- specific signature (p = 0.0007). A nominally significant linear relationship was found for apple juice (p = 0.0117) signature. Graphs for all seven foods can be found in Supplemental File- Relative Metabolism and additional information on methods can be found in Supplemental Fig. S3.

### Association of urinary compounds with blood pressure

The primary finding of the original clinical study was that adopting a DASH-style diet reduced both SBP and DBP, regardless of whether lean pork or chicken/fish was the predominant protein source^11^. Here we analyzed if any urinary FSC were associated with post-diet BP or change in BP. Because none of the urinary FSC had significant associations with post-diet BP, subsequent analysis focused on all urinary compounds, including both FSC and non- FSC compounds. Metabolomics results were first filtered to include only compounds that were present in at least 50% of the urine samples, resulting in 90 urinary compounds for analysis. The abundances of six urinary compounds had nominally significant associations (p < 0.05) with a change in SBP, two of which were also nominally associated with change in DBP (Table 2). For five of the compounds, higher abundances were associated with decreases in BP, on average, as demonstrated through negative effect estimates (Table 2).

**Table 2:** Urinary Compounds Associated with Changes in Blood Pressure Following DASH Diet.

Compound Name	SBP (post – pre)		DBP (post – pre)	
	Effect Estimate	p-value	Effect Estimate	p-value
3-Indolebutyric acid	−0.738	**6.560E-03**	−0.431	**2.246E-02**
1-(beta-D-Ribofuranosyl)−1,4-dihydronicotinamide	0.430	**2.869E-02**	0.117	4.072E-01
Kynuramine	−0.528	**3.435E-02**	−0.319	5.322E-02
Physoperuvine	−5.556	**1.578E-02**	−2.941	**4.593E-02**
265.0971	−0.611	**2.207E-02**	−0.269	1.309E-01
157.0373	−0.669	**1.769E-02**	−0.184	3.524E-01

Linear mixed effects regression was used to determine if any compounds were associated with change in systolic blood pressure (SBP) or change in diastolic blood pressure (DBP). Six compounds were nominally associated with DBP (p < 0.05) of which higher levels of five compounds where associated with a decrease in DBP. Two of these compounds were also associated with a decrease in SBP. For compounds that did not match to a database (i.e. unannotated), only the mass is shown.

The abundances of 16 compounds had nominally significant associations with BP (p < 0.05). While none of the 16 compounds passed multiple testing correction, the abundances of 6 compounds were nominally associated with SBP (p < 0.05) and 5 of these were also nominally associated with DBP (p < 0.05) (Table 3).

**Table 3:** Urinary Compounds Associated with Blood Pressure Pre- and Post- DASH Diet.

Compound Name	Original Data Extraction and Analysis		Following Data Re-extraction		Level of ID*	ID validated using Compound library	Formula Validated using Theoretieal Fragmenation	MS/MS provided
	SBP	DBP	SBP	DBP				
2-Acetyl-3-methylpyrazine	**3.73E-03**	**6.59E-02**	**3.83E-02**	2.05E-01	3			
3-(3-Methylbutylidene)-1(3 H)-isobenzofuranone	1.51E-01	**3.20E-02**	9.47E-01	7.77E-01	3		X	X
3-Indolebutyric acid	4.90E-01	**3.77E-02**	2.90E-01	8.17E-01	3			
2-(3-Methylthiopropyl)malate	**3.95E-02**	**4.62E-02**	5.71E-01	7.66E-01	3		X	X
Bicine	1.68E-01	**1.71E-02**	5.11E-01	2.10E-01	3			
L-Glutamic acid	**3.62E-02**	**1.59E-02**	**1.85E-02**	**1.90E-02**	2	X		X
N-Acetylneuraminic acid	1.32E-01	**2.05E-02**	**4.17E-03**	**1.11E-02**	2	X		X
Potassium gluconate	8.14E-02	**2.37E-02**	8.94E-01	8.26E-01	3			X
VAL-GLU	**5.87E-03**	**3.55E-02**	5.52E-01	5.34E-01	2	X		X
N-(Phenylacetyl)glutamic Acid	**4.05E-02**	1.13E-01	1.03E-01	7.02E-02	3		X	X
73.0264	**1.33E-02**	9.54E-02	9.20E-01	9.46E-01				
121.917	**2.48E-04**	**9.72E-04**	**3.18E-03**	**2.27E-03**				
124.039	**2.10E-03**	**2.24E-03**	3.33E-01	2.96E-01				
238.1336**	**1.95E-02**	**1.75E-02**	1.49E-01	4.15E-01				X
268.1409	**5.72E-05**	**1.82E-04**	**1.86E-03**	**4.63E-03**				X
291.0951	1.32E-01	**2.05E-02**	**4.17E-03**	**1.11E-02**				

Following metabolomics of urine samples, linear regression was used to model association between compounds with SBP or DBP. Seventeen molecules had significant associations (p < 0.05), not adjusting for multiple testing. Putative compound annotations are listed. Where database searching failed to produce a match, the neutral mass and retention time of the detected compound is listed. *The level of confidence according to the Metabolomics Standards Initiative (MSI). Supplemental Figs. S4–S6 show MS/MS spectra for compounds, including matches to MS/MS libraries and CSI:FingerID. Bold indicates nominal significance (p < 0.05) and the highlighted cell passed a Bonferroni corrected threshold for 360 tests (90 metabolites and 4 outcomes; p < 0.00014). To further confirm quantitative results, raw data were re-extracted using a distinct algorithm and peak volumes re-evaluated. One peak was merged during re-extraction (**) resulting in 16 compounds. All signals that have p < 0.05 for the original and re-extracted tests are in the same direction as the original relationship.

## Discussion

Overall, this study demonstrates the utility of a step-wise, nutrimetabolomics approach to evaluating links between diet and health. One novel aspect of this study was the unbiased metabolomics profiling of 12 foods to generate a study-specific compound database. Although a small number of foods were profiled, all of these foods were consumed by all participants and are therefore highly relevant. The inclusion of a study specific database allowed us to assess the overlap of food compounds and to generate food-specific signatures. As expected, PCA results confirmed what is logically assumed, that fruits, vegetables, and meats cluster within their own phyla. Further, acylcarnitines were only detected in dietary protein samples while flavonoids were only found in fruits.

This study also confirmed that FSC, which were unmetabolized, can be detected in 24-hour urine samples using metabolomics. This information is valuable for understanding both an individual’s metabolism and the digestion kinetics of a particular food. Metabolism of a specific food is traditionally measured using a single, known metabolite of that food. Our current metabolomics strategy allowed us to determine how a food-specific signature, derived as an aggregate of a food’s FSC, changed over time. Only grapefruit and apple juice demonstrated differential metabolism signatures, likely due to the small sample size. In addition, in the current study, urinary FSC were observed in a few individuals, limiting our ability to associate FSC with BP. By summing a food’s FSC to create a food-specific signature we were able to include all, even rare, FSC, in our analysis of food metabolism. There are other possible aggregation techniques that could be used (e.g. a summed indicator of the presence of a compound). More research is needed to evaluate aggregation techniques for food-specific signatures.

Our hypothesis that urinary FSC would be significantly associated with BP or change in BP was not supported by the results. This may be due to the low number of FSC seen across multiple individuals, a small effect size for individual foods, or because only unmetabolized compounds were analyzed in this study. If the latter, this would suggest that BP may be more directly related to compounds that have undergone metabolic processing, including Phase I, Phase II, or microbiome-mediated metabolism. This emphasizes a challenge in our step-wise approach and in nutrimetabolomics, whereby it is currently not possible to accurately predict how a food component will be metabolized, either by humans and/or microbes, although new *in silico* tools to predict metabolic products, such as Biotransformer, are becoming available^18^. Although beyond the scope of this study, results from *in silico* metabolism of FSC could be used to further mine the urine datasets for FSC metabolites that relate to BP; however, the potential for false positives may be high.

When all urinary compounds were considered, including FSC and other food-related and endogenous compounds (i.e. non- FSC), 16 were nominally associated with BP and 6 compounds with change in BP. Interestingly, these compounds represented both endogenous-to-human and exogenous compounds. For example, according to HMDB and FooDB, 3-(3-methylbutylidene)-1(3 H)-isobenzofuranone was previously found in green vegetables and wild celery. Celery was included as a snack item in the study diet and its presence in the participant’s urine is plausible. Similarly, 2-acetyl-3-methylpyrazine can be found in cereal and cereal products. Although metabolomics was not performed on any complex foods such as bread, both breads and cereals were included in the participants’ diets. In addition to food-related compounds, known endogenous-to-human compounds including 3-indolebutyric acid, a tryptophan metabolite, L-glutamic acid, N-acetylneuraminic acid, and a dipeptide were associated with BP in this study. Similarly, among compounds associated with changes in BP (Tables 2), 1-(beta-D-ribofuranosyl)-1,4-dihydronicotinamide was identified in milk; physoperuvine was found in fruits; and kynuramine is a metabolite of tryptophan. If replicable, these results suggest that unbiased metabolomics is useful for determining relationships between a wide array of urinary compounds and health indicators such as BP.

There were several limitations to this proof-of-principle study, including a relatively small number of participants and a moderate (~44%) level of compound annotation. A small sample size leads to less stable estimates of association and error in the association models that relate compounds and BP. Additionally, a larger sample size would have potentially enabled us to discover a set of compounds that could be used to define the DASH-style diet and in association analysis with BP. However, because some of the compounds that were significantly associated with BP (Tables 2 and 3) are also associated with the dietary intervention by default, these could potentially be considered “DASH-defining” compounds.

While compounds that were only annotated with mass could still be determined to be statistically significant, not knowing the identity of a compound limits the ability to interpret data. For example, mass-annotated compounds were not considered FSC and not explored further. In addition, determining if compounds were present in a food was a somewhat subjective exercise and is limited by what is currently known. However, this situation is improving as the chemical composition of more foods are elucidated^19^. Because of the large number of compounds surveyed, original citations demonstrating the presence of a compound in a food are not included, although these are listed in the databases used for annotation (Supplemental File_FSC). While the correctness of the database citations is presumed to be high, additional curation is needed to fully confirm the presence of compounds in individual foods. In addition, the accuracy of database annotations is not fully understood, with high potential for artifact when relying on mass alone. The possibility of inaccurate compound annotation emphasizes the need for validation of compounds as potential biomarkers^10^.

A technical limitation is the use of only methanol precipitation during sample preparation of foods which biases the analysis towards hydrophilic compounds and results in relatively low representation of lipids^12^, increasing the potential for overlooking additional biomarkers. Additionally, electrospray ionization was used which is not ideal for detection of non-polar molecules. Thus, a large number of compounds present in foods, particularly plant based foods, were not included in this study. Overall, our results illustrate both the utility and limitation of metabolomics to accurately detect and identify food compounds.

This study demonstrates that the step-wise approach can also be used to discover novel candidate biomarkers of food intake, although limitations exist due to the small number of foods sampled compared to the number of foods available to consumers worldwide. While several compounds had previously been reported in the food of interest, between 9–239 of FSC were found to have been previously reported as present in any food, including foods outside our 12-food study dataset. For example, of the 969 FSC in grapefruit, 239 have been previously found in grapefruit; however, several of these have also been found in other foods, including other citrus foods. Moreover, 344 FSC were found in blueberry and 33 of these are likely to be present in blueberry based on internet searches. For example, epoxy-17-hydroxy-1-oxowitha-3,5,24-trienolide is a constituent of gooseberry and dalpanol O-glucoside is a flavonoid. While considered FSC for the purpose of the current study, these compounds could not be considered specific biomarkers of blueberry intake, but rather of berry consumption in general. As another example, one of the 108 compounds that were unique to apples within our dataset, 4-hydroxydiphenylamine, was previously detected in apples^20^. A similar compound, diphenylamine, is used for control of superficial scald in stored apples^21^. To our knowledge, these compounds have not been detected in foods other than apples and could be considered candidate biomarkers of apple intake, although further validation is required.

In summary, this study demonstrated a step-wise approach to nutrimetabolomics that can be utilized to identify and associate FSC and signatures in biofluids with health indicators. Overall, 824 of the 2009 annotated FSC were discoverable in urine, demonstrating that metabolomics can be used to effectively detect and identify FSC to serve as candidate biomarkers of food intake. Ongoing studies are expanding the food database to include a larger variety of foods and strains and using newly available informatics tools to perform *in silico* metabolism of FSC, to further these initial efforts in nutrimetabolomics.

## Supplementary information

Supporting Information.

Supporting Information 2.

Supporting Information 3.

Supporting Information 4.

Supporting Information 5.

Supporting Information 6.

## Data Availability

This data is available at the NIH Common Fund’s National Metabolomics Data Repository (NMDR) website, the Metabolomics Workbench, https://www.metabolomicsworkbench.org where it has been assigned Project ID PR000843. The data can be accessed directly via it’s Project 10.21228/M8QH5G. NMDR is supported by NIH grant U2C-DK119886.

## References

[1] Claus SP (2014). Development of personalized functional foods needs metabolic profiling. Curr. Opin. Clin. Nutr. Metab. care.

[2] Bouchard-Mercier A, Rudkowska I, Lemieux S, Couture P, Vohl MC (2013). The metabolic signature associated with the Western dietary pattern: a cross-sectional study. Nutr. J..

[3] Rebholz CM, Lichtenstein AH, Zheng Z, Appel LJ, Coresh J (2018). Serum untargeted metabolomic profile of the Dietary Approaches to Stop Hypertension (DASH) dietary pattern. Am. J. Clin. Nutr..

[4] Acar Evrim, Gürdeniz Gözde, Khakimov Bekzod, Savorani Francesco, Korndal Sanne Kellebjerg, Larsen Thomas Meinert, Engelsen Søren Balling, Astrup Arne, Dragsted Lars O. (2018). Biomarkers of Individual Foods, and Separation of Diets Using Untargeted LC-MS-based Plasma Metabolomics in a Randomized Controlled Trial. Molecular Nutrition & Food Research.

[5] Ulaszewska MM (2019). Nutrimetabolomics: An Integrative Action for Metabolomic Analyses in Human Nutritional Studies. Mol. Nutr. Food Res..

[6] Gordon-Dseagu VLZ (2019). The association of sleep with metabolic pathways and metabolites: evidence from the Dietary Approaches to Stop Hypertension (DASH)-sodium feeding study. Metabolomics.

[7] Lenz EM (2004). Metabonomics, dietary influences and cultural differences: a 1H NMR-based study of urine samples obtained from healthy British and Swedish subjects. J. Pharm. Biomed. Anal..

[8] Stella C (2006). Susceptibility of human metabolic phenotypes to dietary modulation. J. Proteome Res..

[9] Pratico G (2018). Guidelines for Biomarker of Food Intake Reviews (BFIRev): how to conduct an extensive literature search for biomarker of food intake discovery. Genes. Nutr..

[10] Gao Q (2017). A scheme for a flexible classification of dietary and health biomarkers. Genes. Nutr..

[11] Sayer RD, Wright AJ, Chen N, Campbell WW (2015). Dietary Approaches to Stop Hypertension diet retains effectiveness to reduce blood pressure when lean pork is substituted for chicken and fish as the predominant source of protein. Am. J. Clin. Nutr..

[12] Yang Y (2013). New sample preparation approach for mass spectrometry-based profiling of plasma results in improved coverage of metabolome. J. Chromatogr. A.

[13] Cruickshank-Quinn CI (2014). Transient and persistent metabolomic changes in plasma following chronic cigarette smoke exposure in a mouse model. PLoS One.

[14] Gagnebin Y (2017). Metabolomic analysis of urine samples by UHPLC-QTOF-MS: Impact of normalization strategies. Anal. Chim. Acta.

[15] Sumner, L. W. *et al*. Proposed Minimum Reporting Standards for Chemical Analysis: Chemical Analysis Working Group (CAWG) Metabolomics Standards Initiative (MSI). (Metabolomics Society, 2007). 10.1007/s11306-007-0082-2 PMC3772505 24039616

[16] Yang X, Neta P, Stein SE (2014). Quality Control for Building Libraries from Electrospray Ionization Tandem Mass Spectra. Anal. Chem..

[17] Shen, H., Dührkop, K., Böcker, S. & Rousu, J. J. B. Metabolite identification through multiple kernel learning on fragmentation trees. **30**, i157-i164 (2014). 10.1093/bioinformatics/btu275 PMC4058957 24931979

[18] Djoumbou-Feunang Y (2019). BioTransformer: a comprehensive computational tool for small molecule metabolism prediction and metabolite identification. J. Cheminform.

[19] Wang M (2016). Sharing and community curation of mass spectrometry data with Global Natural Products Social Molecular Networking. Nat. Biotechnol..

[20] Mattheis JP, Rudell DR (2008). Diphenylamine metabolism in ‘braeburn’ apples stored under conditions conducive to the development of internal browning. J. Agric. Food Chem..

[21] Yihui G (2018). Characterization of laccase from apple fruit during postharvest storage and its response to diphenylamine and 1-methylcyclopropene treatments. Food Chem..

