# Supplementary material for: Nutrimetabolomics reveals food-specific compounds in urine of adults consuming a DASH-style diet
Source: Sci Rep. 2020 Jan 24;10:1157. doi: 10.1038/s41598-020-57979-8 (PMC6981146; doi:10.1038/s41598-020-57979-8)
Supplement: Supplementary file 1 — Supporting Information. [file 41598_2020_57979_MOESM1_ESM.pdf]

Supplemental Information- Figures and Tables

Nutrimetabolomics reveals food-specific compounds in urine of adults consuming a DASH-style diet

Nichole A. Reisdorph, Audrey E. Hendricks, Minghua Tang, Katrina A. Doenges, Richard M. Reisdorph, Brian C. Tooker, Kevin Quinn, Sarah J. Borengasser, Yasmeen Nkrumah-Elie, Daniel N. Frank, Wayne W. Campbell, and Nancy F. Krebs

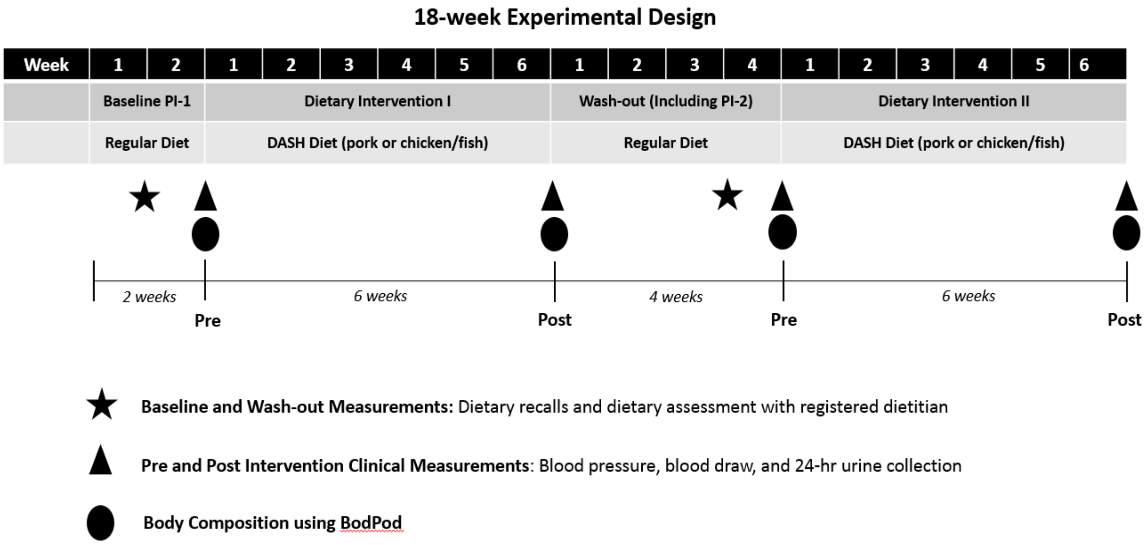

Supplemental Figure S1. Design of the DASH-style diet study

This DASH-style diet based controlled feeding study comprised a cross-over design with washout period. Each participant received both interventions in a random order. Star icons indicate timing of basement and wash-out measurements. Triangles indicate timing of clinical measurements, including 24-hr urine collections. Circles indicate when body composition was assessed.

Hydrophilic spike mix (prepared in 1:1 Methanol:Water)	
**Compound**	**Concentration**
Creatinine-d3	25ug/mL
Lysine-d4	100ug/mL
Valine-d8	200ug/mL
**Lipid Spike mix (prepared in 1:1 Chloroform:Methanol)**	
**Compound**	**Concentration**
FA sat C17:0 (heptadecanoic acid)	200ug/mL
FA unsat C19:1 (cis-10-nonadecenoic acid)	200ug/mL
C17 Ceramide (d18:1/17:0) [N-(heptadecanoyl)-sphing-4-enine]	50ug/mL
17:0 PE (1,2-diheptadecanoyl-sn-glycero-3-phosphoethanolamine)	200ug/mL
15:0 PC (1,2-dipentadecanoyl-sn-glycero-3-phosphocholine)	100ug/mL
Testosterone-d2	100ug/mL
**Hydrophilic Positive Control mix (prepared in 1:1 Methanol:Water)**	
**Compound**	**Concentration**
D-Glucose-$^{13}C_6$	2mg/mL
Alanine-d3	200ug/mL
Methylmalonic acid (d_3-MMA)	200ug/mL
**Lipid Positive control mix (prepared in 1:1 Chloroform:Methanol)**	
**Compound**	**Concentration**
FA sat C15:0 (pentadecanoic acid)	200ug/mL

TG d5-(14:0/16:1(9Z)/14:0)	20ug/mL
15:0 PE (1,2-dipentadecanoyl-sn-glycero-3-phosphoethanolamine)	100ug/mL
17:0 PC (1,2-diheptadecanoyl-sn-glycero-3-phosphocholine)	100ug/mL

Supplementary Table S1. Urine sample preparation standards: Authentic standards were spiked into urine samples to monitor variability in instrument response or batch effects. Concentrations reflect mix levels prior to dilution in urine.

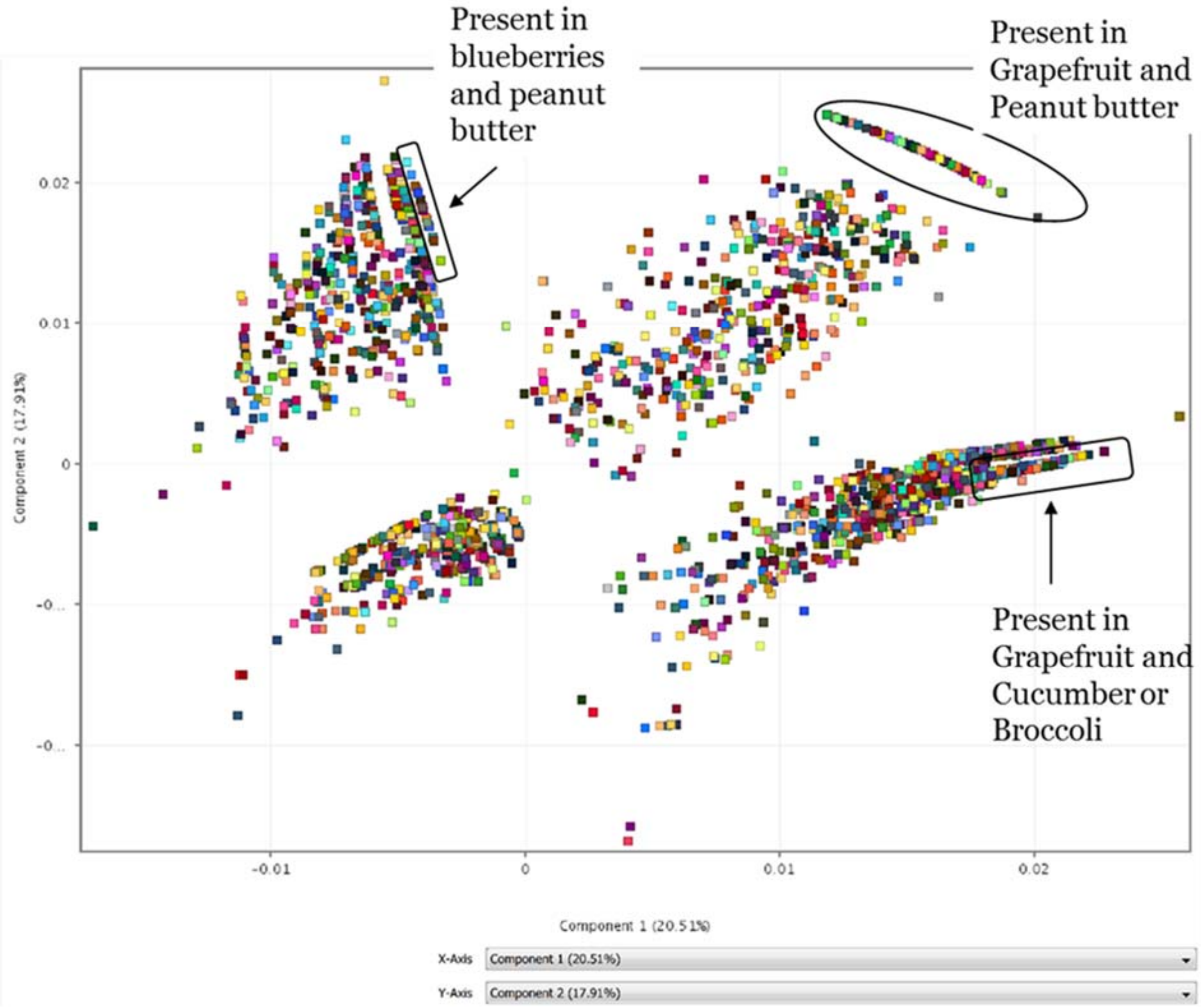

Supplementary Figure S2. PCA loadings plot

Loadings plot showing individual compounds driving the clustering observed in the Figure 2 PCA for the 12 individual food samples in this study.

Supplemental Table 2. Percent overlap of food-derived compounds with urine samples

	**1. Present in ≥1 Pre- and Post-diet urines**	**2. Present in ≥1 Pre- diet urines**	**3. Present in ≥ 1 Post-diet urines**	**4. Present in ≥ 50% Pre- and Post-diet urines**	**5. Present in ≥ 50% Pre-diet urines**	**6. Present in ≥ 50% Post-diet urines**	**7. Present in ≥ 80% Pre- and Post-diet urines**	**8. Present in ≥ 80% Pre-diet urines**	**9. Present in ≥ 80% Post-diet urines**
Number of compounds in the urine dataset being considered [a]	4427	4091	3744	291	352	280	44	49	43
Apples (754)	6.4% (285)	8.2% (336)	18.2% (683)	16.5% (48)	15.3% (54)	17.8% (50)	13.6% (6)	10.2% (5)	18.6% (8)
Apple Juice (702)	7.7% (344)	8.0% (328)	8.0% (302)	20.6% (60)	19.0% (67)	21.1% (59)	13.6% (6)	10.2% (5)	20.9% (9)
Beef (1003)	7.1% (315)	7.0% (286)	7.5% (281)	22.3% (65)	19.3% (68)	25.0% (70)	25% (11)	26.5% (13)	32.5% (14)
Blueberries (1334)	11.5% (512)	11.6% (476)	11.9% (447)	25.0% (73)	23.3% (82)	23.5% (66)	18.3% (9)	14.2% (7)	25.6% (11)
Broccoli (1622)	11.8% (523)	11.9% (488)	12.1% (456)	32.6% (95)	29.5% (104)	35.0% (98)	36.3% (16)	34.7% (17)	46.5% (20)
Chicken (914)	5.4% (240)	5.9% (224)	5.7% (214)	18.9% (55)	17.0% (60)	18.2% (51)	15.9% (7)	16.3% (8)	20.9% (9)
Coffee neat (682)	8.6% (380)	8.8% (359)	8.9% (335)	18.9% (55)	18.5% (65)	20.3% (57)	20.3% (8)	14.2% (7)	23.2% (10)
Cucumber (1645)	12.5% (555)	12.8% (524)	12.8% (481)	31.2% (91)	29.2% (103)	28.9% (81)	27.2% (12)	24.4% (12)	30.2% (13)

Grapefruit (2292)	16.0% (709)	15.9% (652)	16.5% (617)	28.2% (82)	26.7% (94)	29.3% (82)	22.7% (10)	20.4% (10)	37.2% (16)
Peanut Butter (1989)	13.4% (594)	13.6% (557)	13.6% (511)	29.9% (87)	32.1% (113)	26.4% (74)	27.3% (12)	28.6% (14)	28.0% (12)
Pork (730)	5.8% (257)	5.9% (243)	6.3% (235)	22.7% (66)	21.0% (74)	24.5% (69)	25.0% (11)	24.5% (12)	32.6% (14)
Tilapia (531)	3.9% (175)	4.0% (165)	4.4% (165)	17.5% (51)	15.3% (54)	18.6% (52)	11.4% (5)	8.2% (4)	20.9% (9)

Supplemental Table S2. Percent overlap of food-derived compounds with urine samples

Three scenarios were considered to determine what food compounds were also detectable in urine. The first, unnumbered column lists the food name and the number of compounds that were detected in that food. Columns 1-3 compare the overlap of individual food compounds with those compounds found in at least 1 urine sample. Columns 4-6 compare the overlap of individual foods compounds with those compounds found in at least 50% of urine samples. Columns 7-9 compare the overlap of individual food compounds with those compounds found in 80% of urine samples. The percentage of compounds found in common increases as the size of the dataset decreases.

[a]Number of food compounds detected. This is the same value as shown in Table 1, column 2

		Diet Week Day						
**Study Week**	**Diet week**	**1**	**2**	**3**	**4**	**5**	**6**	**7**
**0**	**Pre**	Pre-diet urines collected this week						
**1**	**Pre**	Pre-diet urines collected this week						
**2**	**Pre**							
**3**	**1- Chx/Pork**	1	2	3	4	5	6	7
**4**	**2- Chx/Pork**	8	9	10	11	12	13	14
**5**	**3- Chx/Pork**	15	16	17	18	19	20	21
**6**	**4 - Chx/Pork**	22	23	24	25	26	27	28
**7**	**5 - Chx/Pork**	29 **(4)**	30 **(1)**	31 **(1)**	32 **(4)**	33 **(5)**	34 **(5)**	35 **(2)**
**8**	**6 - Chx/Pork**	36 **(4)**	37 **(1)**	38 **(5)**	39 **(2)**	40 **(3)**	41	42
**No. Urines collected that day**		**8**	**2**	**6**	**6**	**8**	**5**	**2**
**Days since last consumption (eg. Grapefruit)**		**3**	**0**	**1**	**2**	**0**	**1**	**2**
		**Diet Day 1**	**Diet Day 2**	**Diet Day 3**	**Diet Day 4**	**Diet Day 5**	**Diet Day 6**	**Diet Day 7**
	**Grapefruit**		x			x		
	**Apple Juice**		x					
	**Blueberries**		x				x	
	**Broccoli**		x					
	**Apple**			x				
	**Pork Loin**							
	**Cucumber**	x	x					
	**Peanut Butter**	x	x	x			x	
	**Chicken Tenderloin**	x	x	x	2x	x	x	
	**Tilapia**	x	x	x		x	x	
	**Coffee**	x	x	x	x		x	x
	**Beef Tenderloin**							2X

Supplementary Figure S3: Determination of the relative metabolism of food-specific signatures in urine.

Urinary FSC were used to determine if a linear relationship existed between a food-specific signature and time since consumption. The following strategy was used to determine the time since consumption, with the top table comprising the study calendar and the bottom table illustrating days foods were consumed. In the top table, the intervention days, number 1- 42, are highlighted grey or yellow, with urine collection days highlighted yellow. For each DASH-style diet intervention period, urine samples were collected in the pre-diet period and the post-diet period. A rigid schedule was kept for food consumption, with the same foods being consumed on each diet week day; for example, grapefruit was always consumed on Diet Days 2 and 5 (Orange highlighted row). However, the timing of urine collection varied by participant. For example, as shown in the top table above, during Study Week 7, on day 29 of the intervention, urine was collected from 4 participants (the bold number in parentheses). On day 30 of the

intervention, urine was collected from 1 participant while on day 36, urine was collected from 4 participants. Therefore, on Diet Week Day 1, a total of 8 urine samples were collected (Gold highlighted row). Because urines were collected at various Diet Week Days, there was a variable amount of time between urine collection and consumption of a particular food. For example, if urine was collected on Diet Day 1, it had been 3 days since the participants had consumed grapefruit (Blue highlighted row). Using this information, the number of days since last consumption was determined for each food. To determine relative metabolism of foods, a linear mixed effects model was used with individual participant as the random effect, the food specific signature as the outcome, and number of days between when a specific food was consumed and when the urine collection occurred as the predictor. Chx = chicken.

A)

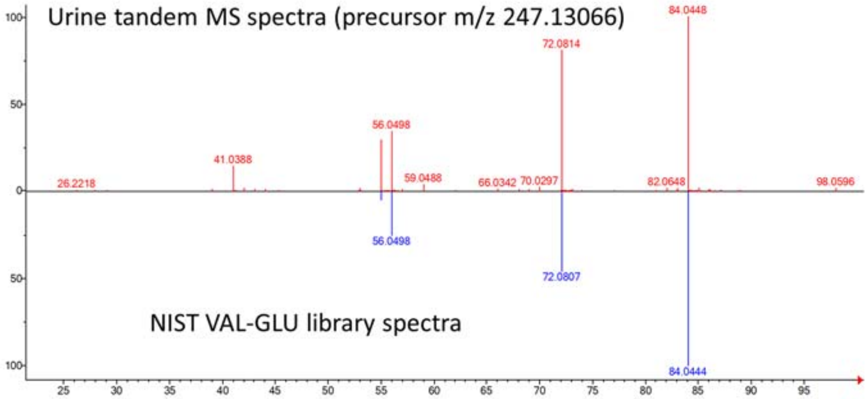

B)

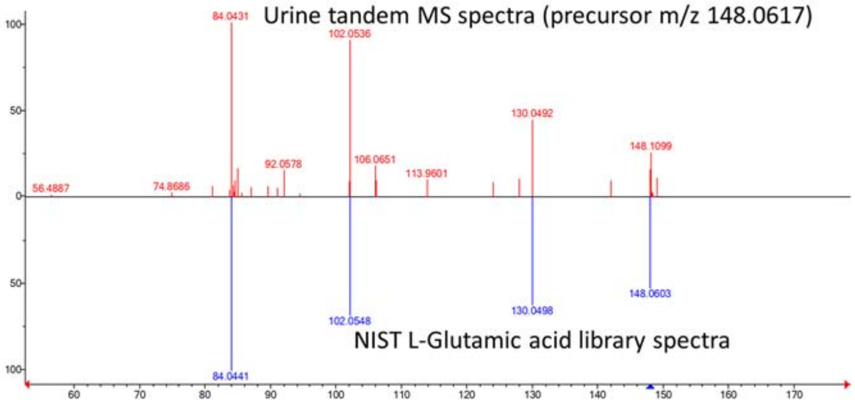

C)

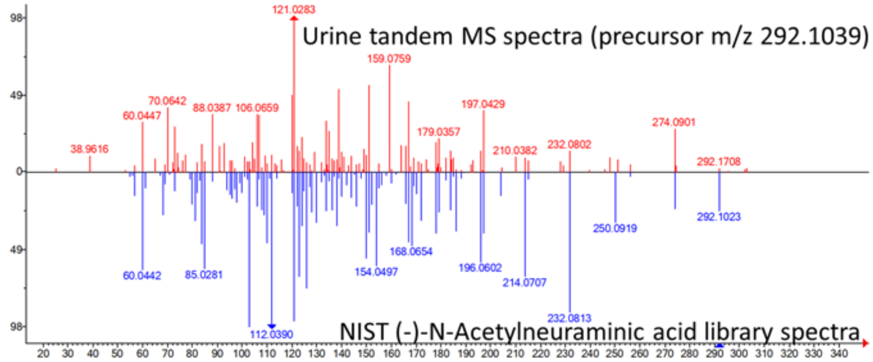

Supplementary Figure S4. Urinary compound matches to the NIST spectral library for the dipeptide VAL-GLU (A), Glutamic acid (B), and (-)-N-Acetylneuraminic (C).

Compounds that correlated with blood pressure (Table 3) were subjected to tandem MS analysis. Spectra were searched using NIST MS Search 2.3 resulting in 3 library matches. The red spectra (top) are the urinary tandem MS spectra and the blue spectra (bottom) are the NIST library matches for the dipeptide VAL-GLU (A), Glutamic acid (B) and (-)-N-Acetylneuraminic (C).

A)

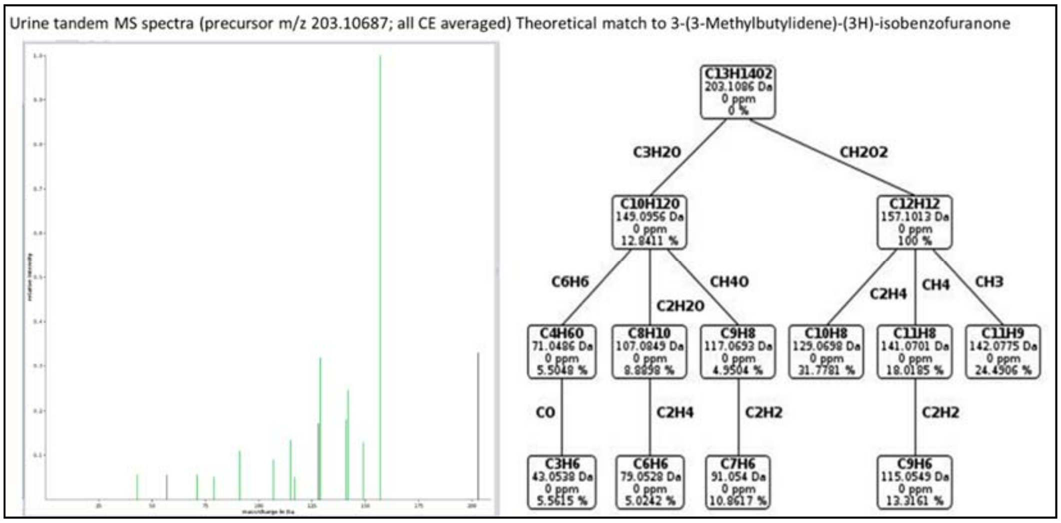

B)

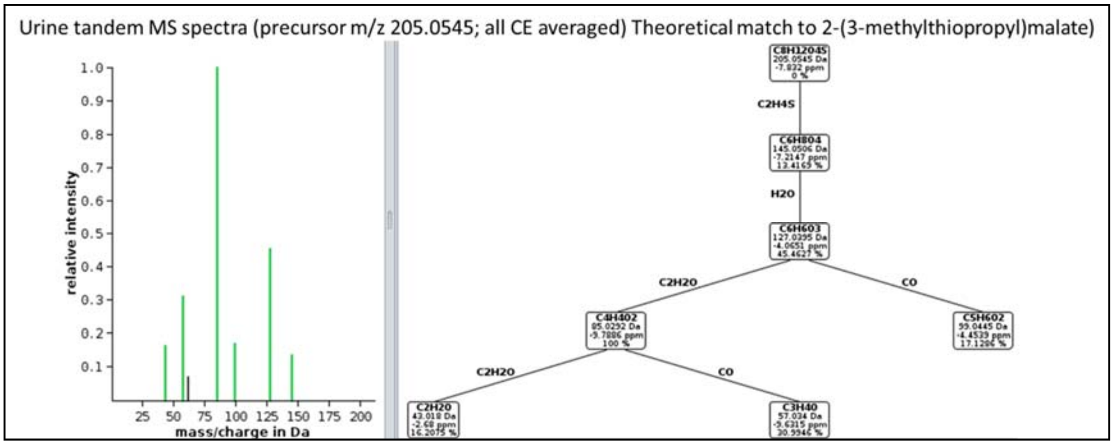

C)

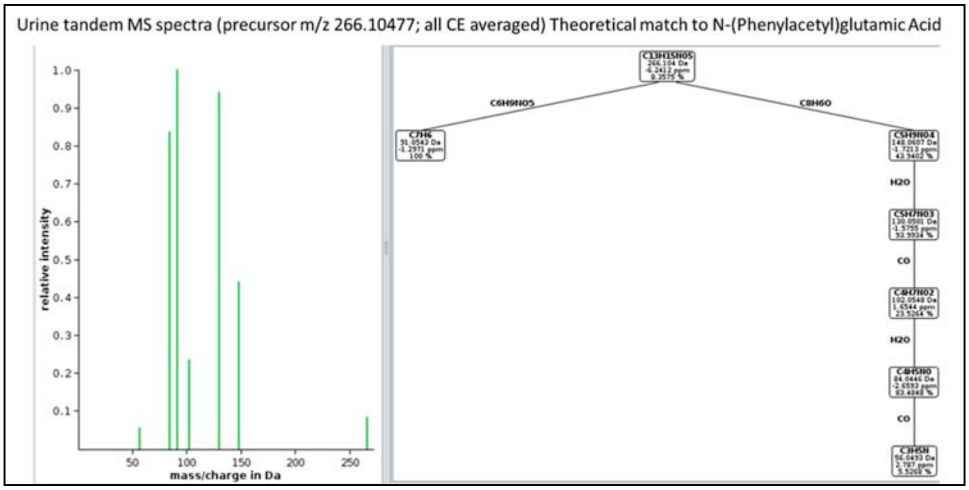

Supplementary Figure S5. Urinary compound matches to CSI:FingerID spectral database for 3-(3-Methylbutylidene)-1(3H)-isobenzofuranone (A), 2-(3-methylthiopropyl)malate (B), and N-(Phenylacetyl)glutamic Acid (C).

Compounds that correlated with blood pressure (Table 3) were subjected to tandem MS analysis. Spectra that did not provide a NIST library hit were re-searched using SIRIUS 4.0.1 to analyze tandem MS fragmentation patterns and CSI:FingerID to search biological molecular structure databases resulting in 3 library matches. The green lines in the spectra (left) represent peaks that are present in the tandem MS spectra that correspond to expected peaks for the predicted chemical formula (right). Urinary compound matches to CSI FingerID spectral database for 3-(3-Methylbutylidene)-1(3H)-isobenzofuranone (A), 2-(3-methylthiopropyl)malate (B), and N-(Phenylacetyl)glutamic Acid (C).

A)

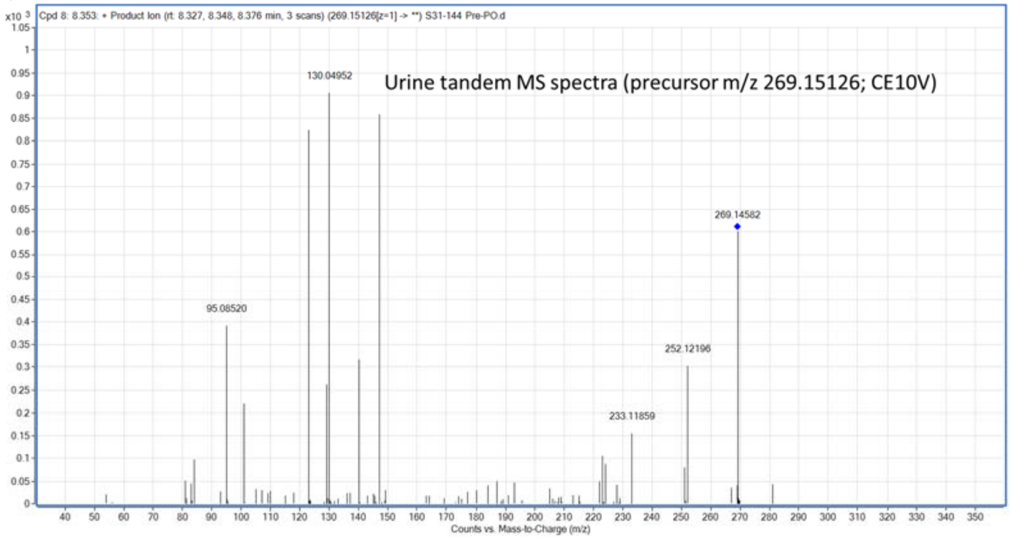
Cpd 8: 8.353: + Product Ion (rt: 8.327, 8.348, 8.376 min, 3 scans) (269.15126[z=1] -> **) S31-144 Pre-PO.d
Urine tandem MS spectra (precursor m/z 269.15126; CE10V)
x10 3
130.04952
269.14582
95.08520
252.12196
233.11859
Counts vs. Mass-to-Charge (m/z)

B)

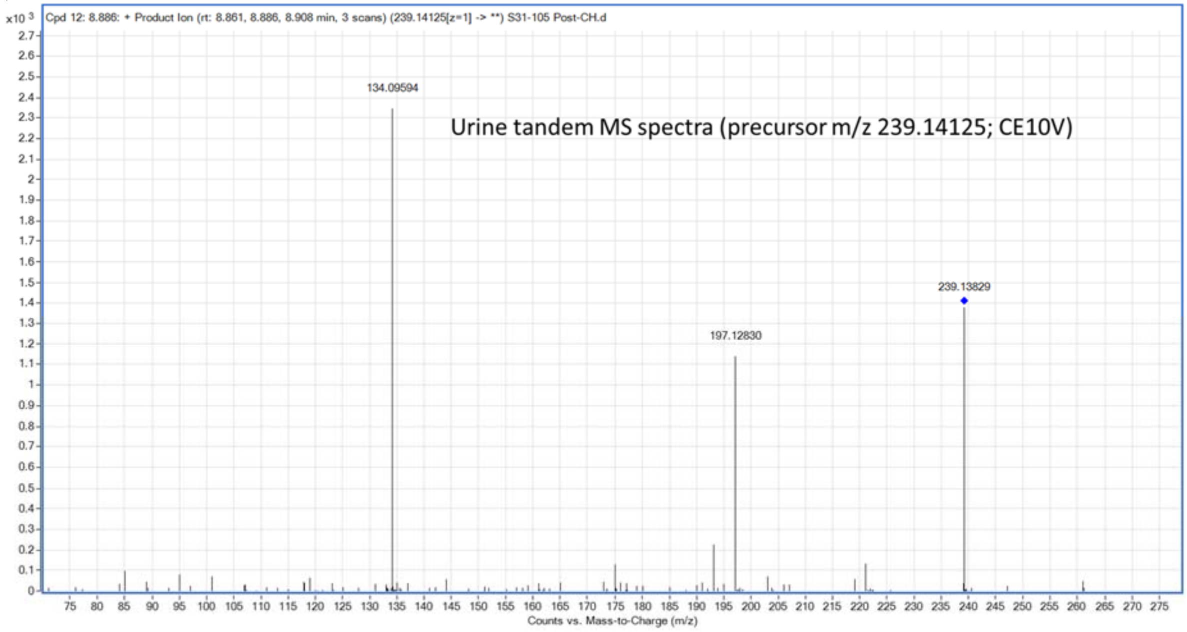
Cpd 12: 8.886: + Product Ion (rt: 8.861, 8.886, 8.908 min, 3 scans) (239.14125[z=1] -> **) S31-105 Post-CH.d
Urine tandem MS spectra (precursor m/z 239.14125; CE10V)
x10 3
134.09594
239.13829
197.12830
Counts vs. Mass-to-Charge (m/z)

C)

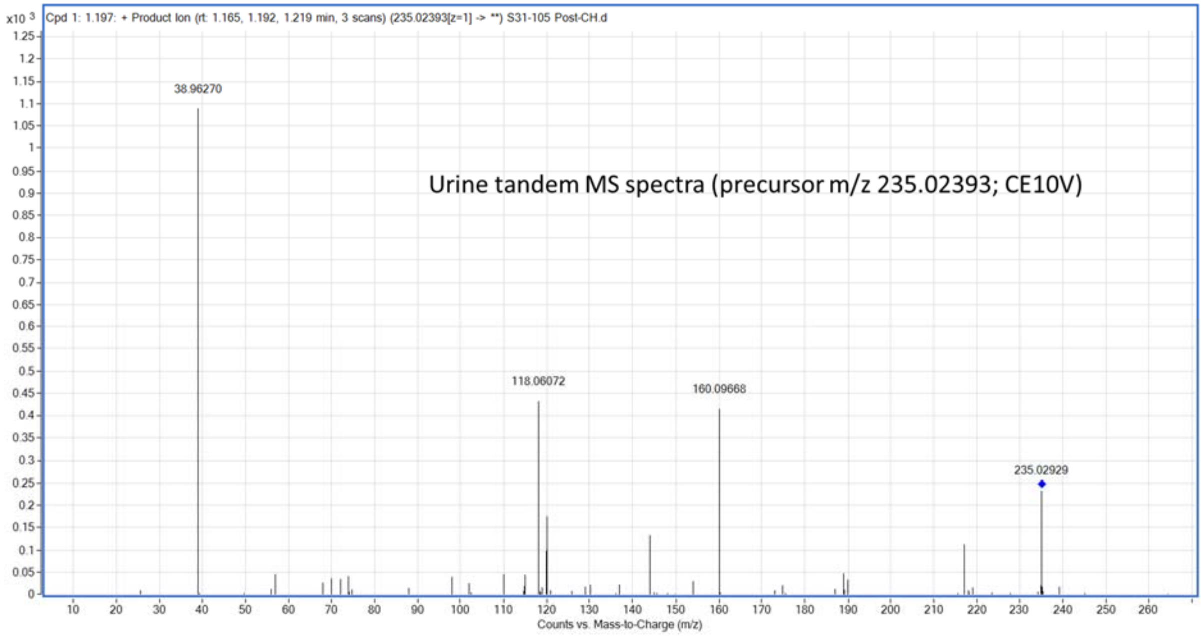

Supplementary Figure S6. Urinary compound spectra that do not match to available compound databases.

Compounds that correlated with blood pressure (Table 3) were subjected to tandem MS analysis. Tandem MS spectra for urinary compounds that did not result in a fragmentation library or theoretical match for m/z 269.1513 (A), m/z 239.1413 (B), and m/z 235.0239 (C).
