# Supplementary material for: Nutrimetabolomics reveals food-specific compounds in urine of adults consuming a DASH-style diet
Source: Sci Rep. 2020 Jan 24;10:1157. doi: 10.1038/s41598-020-57979-8 (PMC6981146; doi:10.1038/s41598-020-57979-8)
Supplement: Supplementary file 2 — Supporting Information 2. [file 41598_2020_57979_MOESM2_ESM.pdf]

Example of a DASH menu's macronutrient summary

Chicken/FISH

DAY	Energy (kcals)	PRO (g)	FAT (g)	CHO (g)	Fiber (g)	SAT FAT (g)	Na (mg)
1	2221.82	100.94	66.29	331.84	31.92	13.98	2415.69
2	2226.33	100.04	64.01	343.28	53.79	16.67	2393.71
3	2172.25	100.13	60.62	317.89	30.33	14.21	2399.11
4	2189.91	101.63	67.46	325.84	55.06	14.73	2436.76
5	2194.55	101.02	62.18	332.56	43.29	19.54	2161.82
6	2225.8	100.18	68.82	320.29	38.87	15.82	2271.88
7	2191.01	100.02	61.67	328.59	36.42	14.06	2494.08
**AVG CHICKEN/FISH**	**2203.095714**	**100.5657143**	**64.43571429**	**328.6128571**	**41.38285714**	**15.57285714**	**2367.578571**

PORK

DAY	Energy (kcals)	PRO (g)	FAT (g)	CHO (g)	Fiber (g)	SAT FAT (g)	Na (mg)
1	2204.49	99.23	65.99	325.39	29.73	16.61	2356.92
2	2202.96	99.42	63.6	339.83	55.77	17.41	2339.22
3	2199.77	99.38	60.65	326.33	35.22	16.93	2368.18
4	2196.84	99.73	66.68	321.17	50.87	18.35	2420.77
5	2155.89	99.8	61.9	321.52	44.94	15.67	2455.34
6	2147.14	96.46	59.62	322.08	40.51	14.88	2391.58
7	2153.48	97.06	58.7	337.24	38.81	12.74	2182.24
**AVG PORK**	**2180.081429**	**98.72571429**	**62.44857143**	**327.6514286**	**42.26428571**	**16.08428571**	**2359.178571**

Example of a DASH-PORK (DASH-P) Menu

Day 1

Foods in Bold Are Provided by the Metabolic Kitchen

**Breakfast**		
Cheerios	3/4	cup
Skim Milk	10	fl oz
Banana	1	medium
Cranberry Juice Cocktail	12	fl oz
Coffee (black) - measure 8 oz	8	fl oz
**Lunch**		
Chef Salad		
Lettuce, Romaine	3	cups
Uncured Ham	**32**	**grams**
Tomato	1	ea
Baby Carrots	10	ea
Cucumber, cubed or diced	1/2	cup
Fat Free Shredded Cheese	22	grams
Croutons	**1**	**packet**
Oil & Vinegar Dressing	**30**	**grams**
Melba Rounds	4	pieces
Fruit Cocktail	1	serving
Diet Soda	12	fl oz
**Mid-Afternoon Snack**		
Honey Multi-Grain Bagel	1/2	bagel
**Country Crock Margarine**	**2**	**ea**
Crystal Light*	8	fl oz
**Dinner**		
Kroger Whole Wheat Hamburger Bun	1	ea
**Pork Loin**	**90**	**grams**
Barbecue Sauce	25	grams
Corn on the Cob	1	ea
**Country Crock Margarine**	**4**	**ea**
Mandrin Oranges	1	ea
Passion Fruit Juice	6	fl oz
**Evening Snack**		
**Popcorn**	**1**	**serving**
Diet Soda	12	fl oz

**Crystal Light can be substituted with water or other beverages sweetened with artificial sweeteners such as Crystal Light or Diet Sodas*
Please refer to the Beverages handout for more information about allowable beverages.

Day 2- DASH-P

Foods in Bold Are Provided by the Metabolic Kitchen

**Breakfast**		
Thomas Hearty Light Multi-Grain English Muffins	1	ea
**Reduced Fat JIF Peanut Butter**	**11**	**grams**
Grapefruit	1/2	large fruit
Apple Juice	8	fl oz
Skim Milk	6	fl oz
Coffee (black) - measure 8 oz	8	fl oz
**Lunch**		
Soft Taco		
Ole Extreme Wellness Whole Wheat Tortilla	1	ea
Ground Pork Taco Filling	40	grams
Lettuce, shredded	1/2	cup
Tomato, diced	1/2	cup
Cheddar Fat Free Shredded Cheese	14	grams
Baked Tostitos Scoops	1	oz
Banana	1	small
Blueberries	1/2	cup
Diet Soda	12	fl oz
**Mid-Afternoon Snack**		
Cherry Tomatoes	5	ea
Baby Carrots	5	ea
Cucumber, sliced with peel	1/2	cup
**Vegetable Dip**	**30**	**grams**
**Dinner**		
**Uncured Ham**	**50**	**grams**
Broccoli	1/2	cup
**Kraft American Cheese**	**9 1/2**	**grams**
Aunt Millie Whole Grain Dinner Roll	1	ea
Country Crock	2	ea
Side Salad		
Lettuce, Shredded	1	cup
Low Sodium Salad Dressing	**15**	**grams**
Vanilla Frozen Yogurt	1/2	cup
Raspberries	1/2	cup
Ice Tea	8	fl oz
Water*	8	oz
**Evening Snack**		
Reduced Fat Vanilla Wafers	5	ea
Water*	8	fl oz

**Water can be substituted with beverages sweetened with artificial sweeteners such as Crystal Light or Diet Sodas.*

Please refer to the Beverages handout for more information about allowable beverages.

**Day 3- DASH-P**		
**Foods in Bold Are Provided by the Metabolic Kitchen**		
**Breakfast**		
Carbmaster Yogurt	1	container (grams)
**Quaker Natural Granola - Oats, Honey & Raisins**	**51**	**grams**
Banana	1	medium
Orange Juice	4	fl oz
Coffee (black) - measure 8 oz	81	cup
**Lunch**		
Tomato Soup (25% less sodium)	1/3	cup
Low Sodium Oyster Crackers	5	ea
Healthy Life 100% Whole Wheat Bread	1	slice
**Uncured Ham**	**38**	**grams**
Reduced Fat Cheddar Cheese	22	grams
Country Crock	3	each
Apple	1	ea
Crystal Light*	8	fluid oz
**Mid-Afternoon Snack**		
Celery strips, 4" long	8	ea
**Reduced Fat JIF Peanut Butter**	**22**	**grams**
Diet Soda	1	can
**Dinner**		
**Pork Loin**	**70**	**grams**
Gravy, turkey or pork, in jar	23	grams
Healthy Life 100% Whole Wheat Bread	1	slice
**Country Crock Margarine**	**5**	**ea**
**Jelly, reduced sugar**	**1**	**packet**
Sweet Potatoes	1/2	cup
Mini Marshmallows	30	ea
**Green Beans**		
**Mixed Fruit**	**1/2**	**cup (1 fruit cup)**
Ice Tea	8	fl oz
**Evening Snack**		
**Quaker Chocolate Chunk Granola Bar**	**1**	**ea**
Water*	8	fl oz
**Water can be substituted with beverages sweetened with artificial sweeteners such as Crystal Light or Diet Sodas.*		
Please refer to the Beverages handout for more information about allowable beverages.		

**Day 4- DASH-P**		
**Foods in Bold Are Provided by the Metabolic Kitchen**		
**Breakfast**		
Cheerios	1	cup
1% Milk	10	fl oz
Peaches	1/2	cup
Orange Juice	6	fl oz
Coffee (black) - measure 8 oz	1	cup
**Lunch**		
Pork Quesadilla		
Thomas Hearty Multi-Grain Light English Muffins	1	ea
Pork Loin	**20**	**grams**
Reduced Fat Cheddar Cheese	22.6	grams
Onion, chopped	1/4	cup
Green Pepper, chopped	1/2	cup
Red Pepper, chopped	1/2	cup
Banana	1	medium
Cherry Tomatoes	10	ea
Baby Carrots	10	ea
**Vegetable Dip**	**32**	**grams**
Diet Soda	1	can
Water*	8	fluid oz
**Mid-Afternoon Snack**		
Carbmaster Yogurt	1/2	container
**Quaker Natural Granola**	**25**	**grams**
Crystal Light*	8	fl oz
**Dinner**		
Pork Waldroff Salad		
**Pork Loin**	**65**	**grams**
Romaine Lettuce, Shredded	2 1/2	cups
Parmesan Cheese	2	tsp
Green Beans, string	1/2	cup
Mushrooms	1/2	cup
Pears	1	cup
**Low Sodium Salad Dressing**	**10**	**grams**
Peas	1/2	cup
**Country Crock Margarine**	**3**	**ea**
Melba Rounds	4	ea
Apple Cinnamon Fruit Crisp	1	ea
Water*	8	fl oz
**Evening Snack**		
**Popcorn**	**1**	**serving**
Diet Soda (any flavor)	1	12 oz can
**Water can be substituted with beverages sweetened with artificial sweeteners such as Crystal Light or Diet Sodas.*		
Please refer to the Beverages handout for more information about allowable beverages.		

**Day 5- DASH-P**		
**Foods in Bold Are Provided by the Metabolic Kitchen**		
**Breakfast**		
Quaker Instant Oatmeal (any fruit & cream variety)	1	ea
Country Crock	2	ea
Grapefruit	1/2	medium fruit
Skim Milk	11	fluid oz
**Lunch**		
Pork Tenderloin Sandwich:		
Kroger Whole Wheat Hamburger Bun	1	ea
Pork Loin	**50**	**grams**
Lettuce, inner leaf	2	ea
Tomato, slice	1	ea
Light Mayonnaise	**2**	**Tbsp**
Lay's Lightly Salted Potato Chips	28	gram
Kiwi Fruit	1	ea
Crystal Light	1	packet
**Mid-Afternoon Snack**		
Green Pepper, sliced	3/4	cup
Red Pepper, sliced	3/4	cup
**Vegetable Dip**	**25**	**grams**
Diet Soda (any flavor)	1	can
**Dinner**		
Pizza:		
Thomas Hearty Multi-Grain Light English Muffins	1	ea
Pizza Sauce	3/4	cup
Uncured Ham	**28**	**grams**
Pineapple	1/2	cup
Fat Free Mozzarella Cheese	1/3	cup
Frozen Yogurt	1/2	cup
Raspberries	1/2	cup
Frozen Fruit Bar	1	ea
Water*	8	fl oz
**Evening Snack**		
**Quaker Chocolate Chunk Granola Bar**	**1**	**ea**
**Water can be substituted with beverages sweetened with artificial sweeteners such as Crystal Light or Diet Sodas.*		
Please refer to the Beverages handout for more information about allowable beverages.		

Day 6- DASH-P

Foods in Bold Are Provided by the Metabolic Kitchen

**Breakfast**		
Kellogg's Eggo Golden Oat Waffles	2	ea
**Country Crock Margarine**	**2**	**ea**
Syrup	1/2	Tbsp.
Blueberries	1/2	cup
Skim Milk	10	fluid oz
Coffee (black) - measure 8 oz	1	cup
**Lunch**		
Ham & Veggie Wrap		
Ole Extreme Wellness Whole Wheat Tortilla	1	ea
Uncured Ham	**42**	**grams**
Lettuce, shredded	1/2	cup
Tomatoes, diced	1/2	cup
Light Mayonnaise	1	packet
Melba Rounds	8	ea
Banana	1	medium
Crystal Light*	1	packet
**Mid-Afternoon Snack**		
Celery, 4 inch strips	6	ea
**Reduced Fat Peanut Butter**	**1**	**Tbsp**
Crystal Light*	1	packet
**Dinner**		
Southwestern Pork Salad		
Lettuce, romaine, shredded	2	cups
Canola Oil	5	grams
Pork Loin	**60**	**grams**
Red Pepper, chopped	1/4	cup
Green Pepper, chopped	1/4	cup
Onions, chopped	1/4	cup
Mild Cheddar Cheese, Shredded	15	grams
Salsa	30	grams
Corn	1/2	cup
Baked Tostitos Scoops	1	oz
Mandarin Oranges	1/2	cup (1 fruit cup)
Peach Fruit Crisp	1	ea
Ice Tea	12	fl oz
**Evening Snack**		
**Popcorn**	**1**	**serving**
Diet Soda	1	can

**Water can be substituted with beverages sweetened with artificial sweeteners such as Crystal Light or Diet Sodas.*
Please refer to the Beverages handout for more information about allowable beverages.

**Day 7- DASH-P**		
**Foods in Bold Are Provided by the Metabolic Kitchen**		
**Breakfast**		
Honey Multi-Grain Bagel	1	ea
**Country Crock Margarine**	**4**	**ea**
**Jelly, reduced sugar**	**2**	**packets**
Strawberries	1/2	cup
Skim Milk	4	fluid oz
Coffee (black) - measure 8 oz	1	cup
**Lunch**		
Asain Beef Stir-Fry		
Beef Tenderloin	**22**	**grams**
Canola Oil	9	grams
Broccoli	1	cup
Mushrooms	1/2	cup
Corn	1/2	cup
Snowpeas	50	grams
Soy Sauce	0..6	Tbsp
Brown Rice	3/4	cup
Peas & Carrots	1/2	cup
**Pineapple**	**1/2**	**cup**
Rold Gold Tiny Twist Pretzels	13	each
Apple	1	medium
Water*	8	fl oz
**Mid-Afternoon Snack**		
**Light Ice Cream**	**1**	**serving**
1% Milk	1/2	cup (140 grams)
Hot Fudge or Hot Caramel	2	Tbsp
**Dinner**		
Hamburger patty, 90% lean ground beef, RAW weight**	73	grams
Kroger Whole Wheat Hamburger Bun	1	ea
Kroger 2% Fat American Singles, 21 g ea	1/2	ea
Van Camp's Pork & Beans	1/4	cup
Green Grapes	20	ea
Water*	8	fl oz
**Water can be substituted with beverages sweetened with artificial sweeteners such as Crystal Light or Diet Sodas.*		
Please refer to the Beverages handout for more information about allowable beverages.		
**73 grams of RAW 90% lean ground burger equals 58 grams of COOKED 90% lean ground burger.		

Example of a DASH-CHICKEN-FISH Menu

Day 1

Foods in Bold Are Provided by the Metabolic Kitchen

**Breakfast**		
Cheerios	3/4	cup
1% Milk	16	fl oz (2 cups)
Banana	1	large
Ocean Spray Light Cranberry Juice	6	fl oz (3/4 cup)
Coffee (black) - measure 8 oz	8	fl oz (1 cup)
**Lunch**		
Chef Salad		
Lettuce, Romaine	3	cups
Chicken Breast Tenderloin	**85**	**grams**
Tomato, roma	1	ea
Baby Carrots	10	ea
Cucumber, cubed or diced	1	cup
Mild Shredded Cheese	1/4	cup
Croutons	**1**	**packet**
Low Sodium Salad Dressing	**40**	**grams**
Melba Rounds	4	pieces
Diet Soda	12	fl oz
**Mid-Afternoon Snack**		
Honey Multi-Grain Bagel	1/2	bagel
**RF Peanut Butter**	**10**	**grams**
Crystal Light*	8	fl oz
**Dinner**		
Kroger Whole Wheat Hamburger Bun	1	ea
**Tilapia Fillet**	**95**	**grams**
**Light Mayo**	**1**	**Tbsp**
Corn on the Cob	1	ea
**Country Crock Margarine**	**4**	**ea**
Mandarin Oranges (drink juice)	1 1/4	cup
Ocean Spray Light Cranberry Juice	14	fl oz (1 3/4 cups)
1% Milk	8	fl oz (1 cup)
**Evening Snack**		
**Popcorn**	**1**	**serving**
Diet Soda	12	fl oz

**Crystal Light can be substituted with water or other beverages sweetened with artificial sweeteners such as Crystal L*
Please refer to the Beverages handout for more information about allowable beverages.

Day 2- DASH-C/F

Foods in Bold Are Provided by the Metabolic Kitchen

**Breakfast**		
Thomas Hearty Light Multi-Grain English Muffins	2	ea
**Reduced Fat JIF Peanut Butter**	**18**	**grams**
Grapefruit	1/2	large fruit
Apple Juice	6	fl oz (3/4 cup)
1% Milk	8	fl oz (1 cup)
Coffee (black) - measure 8 oz	8	fl oz (1 cup)
**Lunch**		
Soft Taco		
Ole Extreme Wellness Whole Wheat Tortilla	1	ea
Chicken Breast Tenderloin	**65**	**grams**
Lettuce, shredded	1/2	cup
Tomato, diced	1/2	cup
Fat Free Cheddar Shredded Cheese	1/4	cup
Baked Tostitos Scoops	20	ea
No Salt Salsa	2	Tbsp
Banana	1	medium
Blueberries	1	cup
Diet Soda	12	fl oz
**Mid-Afternoon Snack**		
Cherry Tomatoes	4	ea
Baby Carrots	5	ea
Cucumber, sliced with peel	1/2	cup
**Vegetable Dip**	**25**	**grams**
**Dinner**		
**Tilapia Fillet**	**70**	**grams**
Broccoli	1/2	cup
Kraft American Cheese Slices (19 g each)	1	each
King's Hawaiian Honey Wheat Roll	1	ea
**Country Crock Margarine**	**1**	**ea**
Side Salad		
Lettuce, Shredded	1	cup
Low Sodium Salad Dressing	**30**	**grams**
Raspberries	1	cup
Ice Tea	8	fl oz
**Evening Snack**		
Reduced Fat Vanilla Wafers	8	ea
1% Milk	10	fl oz (1 1/4 cup)
Water*	8	fl oz

**Day 3- DASH-C/F**		
**Foods in Bold Are Provided by the Metabolic Kitchen**		
**Breakfast**		
Kroger Carbmaster Yogurt	1	container
**Quaker Natural Granola - Oats, Honey & Raisins**	**25**	**grams**
Banana	1	large
Orange Juice	8	fl oz (1 cup)
1% Milk	6	fl oz (3/4 cup)
Coffee (black) - measure 8 oz	1	cup
**Lunch**		
Tomato Soup (25% less sodium)	1/4	cup
Low Sodium Oyster Crackers	10	ea
Healthy Life 100% Whole Wheat Bread	1	slices
**Tilapia Fillet**	**120**	**grams**
**Country Crock Margarine**	**5**	**each**
Apple	1	large
Apple Pear Fruit Crisp	1	each
Crystal Light*	8	fluid oz
**Mid-Afternoon Snack**		
Celery strips, 4" long	8	ea
**JIF Peanut Butter**	**25**	**grams**
Diet Soda	1	can
**Dinner**		
**Chicken Breast Tenderloin**	**125**	**grams**
Gravy, turkey or pork, in jar	1	Tbsp
Healthy Life 100% Whole Wheat Bread	1	slices
**Country Crock Margarine**	**6**	**ea**
**Jelly, reduced sugar**	**2**	**packet**
Sweet Potato (cooked, without skin)	1	each
**Mini Marshmallows**	**40**	**ea**
Green Beans	1/2	cup
Mixed Fruit	1	cup
1% Milk	8	fl oz (1 cup)
Ice Tea	8	fl oz
**Evening Snack**		
**Quaker Chocolate Chunk Granola Bar**	**2**	**ea**
Water*	8	fl oz
**Water can be substituted with beverages sweetened with artificial sweeteners such as Crystal Light or Diet Sodas.*		
Please refer to the Beverages handout for more information about allowable beverages.		

Day 4- DASH-C/F

Foods in Bold Are Provided by the Metabolic Kitchen

**Breakfast**		
Cheerios	1	cup
1% Milk	12	fl oz (1 1/2 cups)
Peaches	1	cup
Orange Juice	8	fl oz (1 cup)
Coffee (black) - measure 8 oz	1	cup
**Lunch**		
Chicken Quesadilla		
Thomas Hearty Multi-Grain Light English Muffins	2	ea
Chicken Breast Tenderloin	**60**	**grams**
Reduced Fat Cheddar Cheese	1/4	cup
Onion, chopped	1/4	cup
Banana	1	large
Cherry Tomatoes	8	ea
Baby Carrots	8	ea
**Vegetable Dip**	**30**	**grams**
Diet Soda	1	can
Water*	8	fluid oz
**Mid-Afternoon Snack**		
Carbmaster Yogurt	1	container
**Quaker Natural Granola**	**15**	**grams**
Crystal Light*	8	fl oz
**Dinner**		
Chicken Waldroff Salad		
**Chicken Breast Tenderloin**	**92**	**grams**
Romaine Lettuce, Shredded	2 1/2	cups
Green Beans	1	cup
Mushrooms	1/2	cup
Pears	1	cup
**Low Sodium Salad Dressing**	**50**	**grams**
Peas	1/2	cup
**Country Crock Margarine**	**5**	**ea**
Melba Rounds	4	ea
Apple Pear Fruit Crisp	1	ea
Water*	8	fl oz
**Evening Snack**		
**Popcorn**	**1**	**serving**
Diet Soda (any flavor)	1	12 oz can

**Day 5- DASH-C/F**		
**Foods in Bold Are Provided by the Metabolic Kitchen**		
**Breakfast**		
Quaker Instant Oatmeal (any fruit & cream variety)	1	ea
**Country Crock Margarine**	**3**	**ea**
Grapefruit	1/2	large fruit
1% Milk	14	fl oz (1 3/4 cups)
**Lunch**		
Chicken Sandwich:		
Kroger Whole Wheat Hamburger Bun	1	ea
Chicken Breast Tenderloin	**118**	**grams**
Lettuce, inner leaf	2	ea
Tomato, slice	1	ea
Light Mayonnaise	**2**	**Tbsp**
Lay's Lightly Salted Potato Chips	15	ea
Kiwi Fruit	1	ea
Crystal Light	1	packet
**Mid-Afternoon Snack**		
Baby Carrots, raw	10	each
**Vegetable Dip**	**25**	**grams**
Edy's Frozen Fruit Bar	1	ea
Diet Soda (any flavor)	1	can
**Dinner**		
Pizza:		
Thomas Hearty Multi-Grain Light English Muffins	1	ea
Pizza Sauce	3/4	cup
Tilapia Fillet	**68**	**grams**
Pineapple	1	cup
Fat Free Mozzarella Cheese	1/3	cup
Frozen Yogurt	3/4	cup
Raspberries	3/4	cup
Water*	8	fl oz
**Evening Snack**		
**Quaker Chocolate Chunk Granola Bar**	**1**	**ea**
1% Milk	8	fl oz (1 cup)
Apple	1	large
**Water can be substituted with beverages sweetened with artificial sweeteners such as Crystal Light or Diet Sodas.*		
Please refer to the Beverages handout for more information about allowable beverages.		

**Day 6- DASH-C/F**		
**Foods in Bold Are Provided by the Metabolic Kitchen**		
**Breakfast**		
Kellogg's Eggo Golden Oat Waffles	2	ea
**Country Crock Margarine**	**2**	**ea**
Syrup	2	Tbsp.
Blueberries	3/4	cup
1% Milk	12	fl oz (1 3/4 cups)
Banana	1	large
Coffee (black) - measure 8 oz	1	cup
**Lunch**		
Fish & Veggie Wrap		
Ole Extreme Wellness Whole Wheat Tortilla	1	ea
Tilapia Fillet	**57**	**grams**
Lettuce, shredded	1	cup
Tomatoes, diced	1	cup
Light Mayonnaise	**2**	**tsp**
Melba Rounds	4	ea
Strawberries	1 1/2	cups
1% Milk	8	fl oz (1 cup)
Crystal Light*	1	packet
**Mid-Afternoon Snack**		
Celery, 4 inch strips	8	ea
**JIF Peanut Butter**	**32**	**g**
Crystal Light*	1	packet
**Dinner**		
Southwestern Chicken Salad		
Lettuce, romaine, shredded	2	cups
Canola Oil	1	Tbsp
Chicken Breast Tenderloin	**86**	**grams**
Onions, chopped	1/4	cup
Reduced Fat Cheddar Cheese, Shredded	1/2	cup
Salsa	1/4	cup
Corn	1/2	cup
Baked Tostitos Scoops	20	ea
Mandarin Oranges (drink juice)	1	cup
Peach Fruit Crisp	1	ea
1% Milk	8	fl oz (1 cup)
Edy's Frozen Fruit Bar	1	each
Ice Tea	12	fl oz
**Evening Snack**		
**Popcorn**	**1**	**serving**
Diet Soda	1	can

**Day 7- DASH-C/F**		
**Foods in Bold Are Provided by the Metabolic Kitchen**		
**Breakfast**		
Honey Multi-Grain Bagel	1	ea
**Country Crock Margarine**	**3**	**ea**
**Jelly, reduced sugar**	**3**	**packets**
Strawberries	1	cup
1 % Milk	10	fl oz (1 1/4 cups)
Coffee (black) - measure 8 oz	1	cup
**Lunch**		
Asian Beef Stir-Fry		
Beef Tenderloin	**38**	**grams**
Canola Oil	1	Tbsp
Broccoli	1	cup
Mushrooms	1/2	cup
Corn	1/2	cup
Snowpeas	1/2	cup
Soy Sauce	2	tsp
Brown Rice	1 1/4	cup
Peas & Carrots	1/2	cup
Pineapple	1	cup
Water*	8	fl oz
**Mid-Afternoon Snack**		
Kroger Carbmaster Yogurt (any flavor)	1	container
**Quaker Natural Granola**	**20**	**g**
**Dinner**		
**Beef Tenderloin**	**67**	**g**
Ore Ida Steam & Cut Mash Potatoes	1 1/3	cup
Gravy, turkey or pork, in jar	1/4	cup
King's Hawaiian Honey Wheat Rolls	1	ea
**Country Crock Margarine**	**5**	**ea**
Peas	1/2	cup
Kroger Fat Free Cottage Cheese	1/3	cup
Peaches	1	cup
Edy's Frozen Fruit Bar	1	each
Ice Tea	8	fl oz
**Evening Snack**		
**Quaker Chocolate Chunk Granola Bar**	**1**	**ea**
**Water can be substituted with beverages sweetened with artificial sweeteners such as Crystal Light or Diet Sodas.*		
Please refer to the Beverages handout for more information about allowable beverages.		
