# Supplementary material for: Nutrimetabolomics reveals food-specific compounds in urine of adults consuming a DASH-style diet
Source: Sci Rep. 2020 Jan 24;10:1157. doi: 10.1038/s41598-020-57979-8 (PMC6981146; doi:10.1038/s41598-020-57979-8)
Supplement: Supplementary file 4 — Supporting Information 4. [file 41598_2020_57979_MOESM4_ESM.pdf]

Supplemental File: Relative Metabolism of Food-Specific Compounds (FSCs) Detected in Urine

Following analysis of food samples using LC/MS, food compounds were categorized as FSCs as described in the manuscript. These FSCs were filtered based on their presence in at least 1 urine sample (see Table 1, Column 4 in the manuscript). The resulting "food-specific signatures" were used to determine the relative metabolism for each food. A "signature" is defined in this study as an aggregate of all FSC for a food that were also detected in urine and includes the total sum of that food's FSC abundance values.

The table shows p-values for each of the food signatures tested. Compounds passing significance were significant after adjusting for the number of foods tested. Graphs are included for each food with the name of the food listed on the y-axis.

FOOD	Days included	ANOVA.p.value	Continuous p.value
**grapefruit**	**All Days**	**0.0058**	**0.0007**
grapefruit	Day 2	0.0050	0.0002
grapefruit	Day 5	0.0050	0.7691
**apple.juice**	**All Days**	**0.1460**	**0.0117**
**blueberries**	**All Days**	**0.7263**	**0.9842**
blueberries	Day 2	0.8257	0.3170
blueberries	Day 6	0.8257	0.5356
**broccoli**	**All Days**	**0.7817**	**0.1842**
**cucumber**	**All Days**	**0.4594**	**0.4849**
cucumber	Day 1	0.1178	0.7564
cucumber	Day 2	0.1178	0.0404
**peanut.butter**	**All Days**	**0.2588**	**0.6440**
peanut.butter	Day 1	0.0771	0.8440
peanut.butter	Day 2	0.0771	0.0252
peanut.butter	Day 3	0.0771	0.4052
**beef.tenderloin**	**All Days**	**0.9791**	**0.7886**

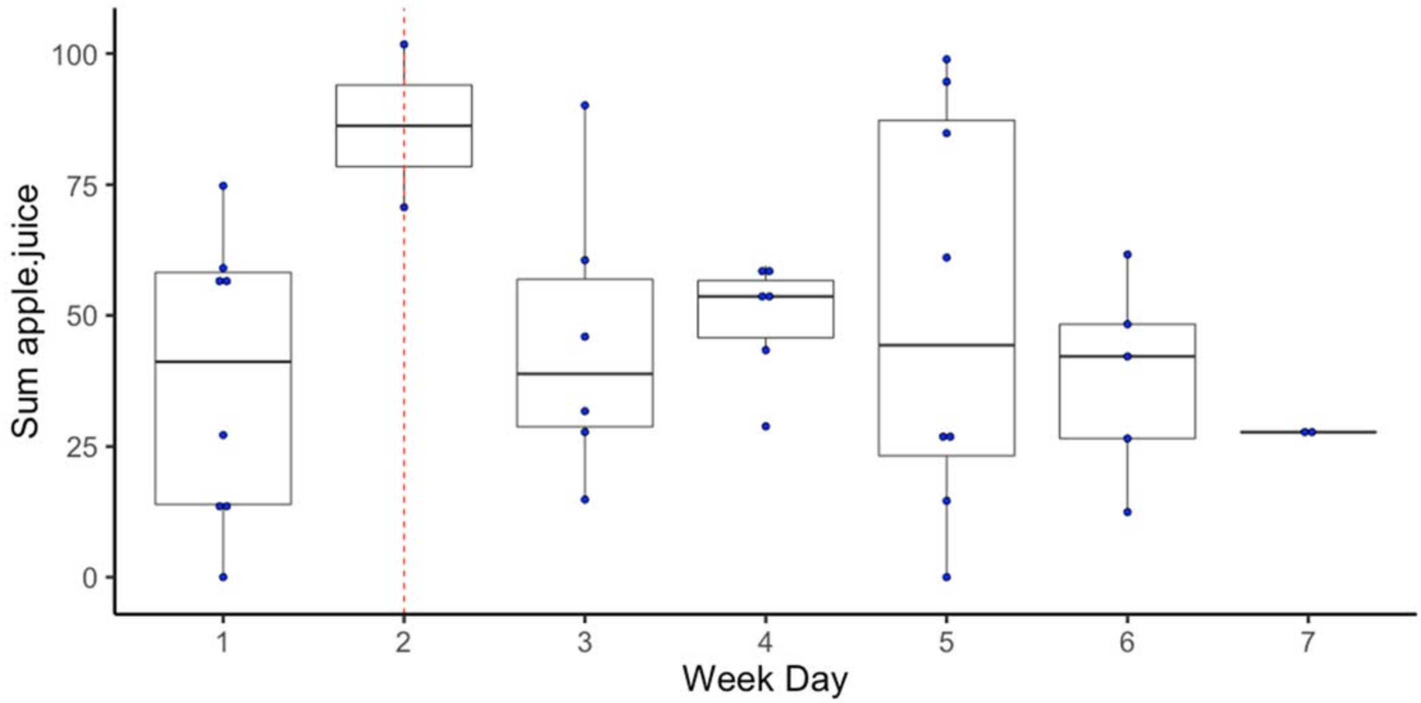

100
75
50
25
0
Sum apple.juice
1
2
3
4
5
6
7
Week Day

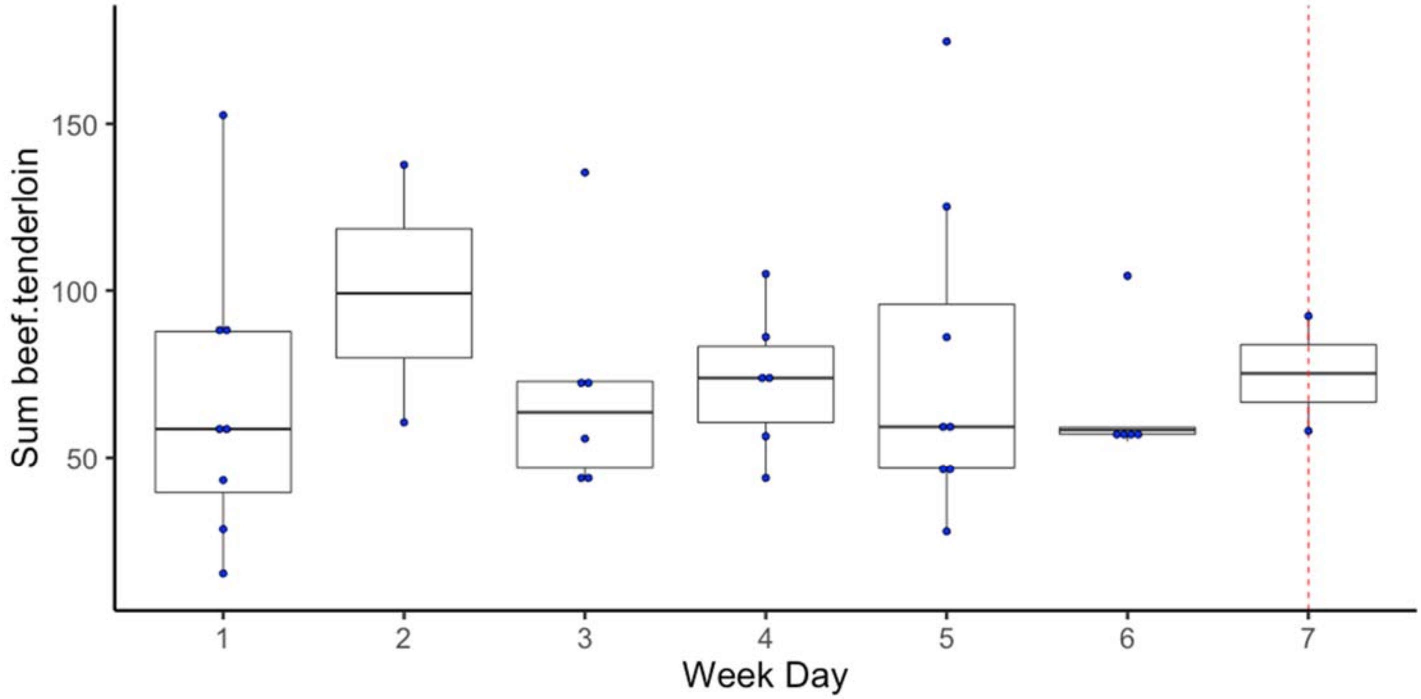

150
100
50
Sum beef.tenderloin
1
2
3
4
5
6
7
Week Day

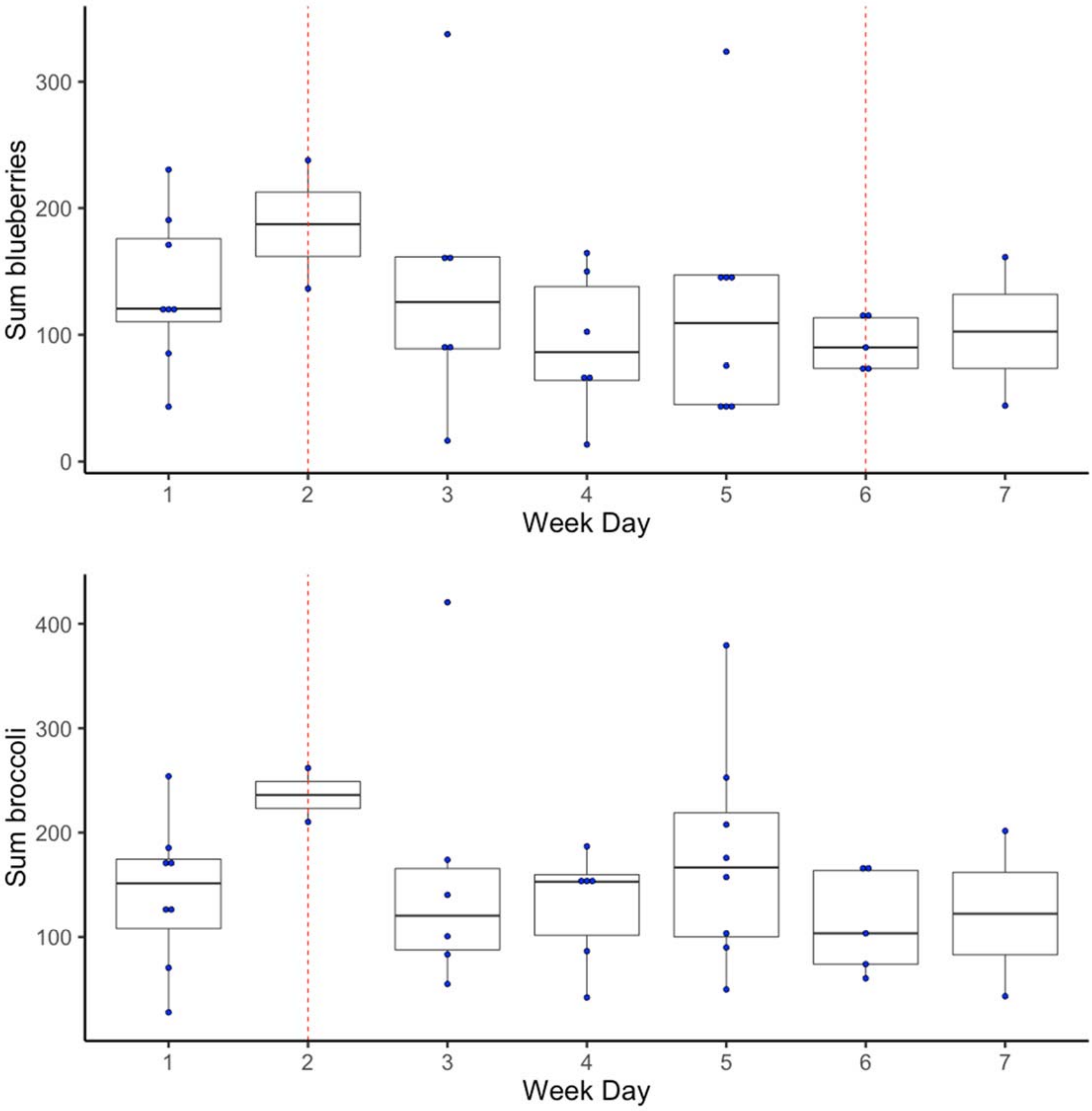
Sum blueberries
300
200
100
0
1
2
3
4
5
6
7
Week Day
Sum broccoli
400
300
200
100
1
2
3
4
5
6
7
Week Day

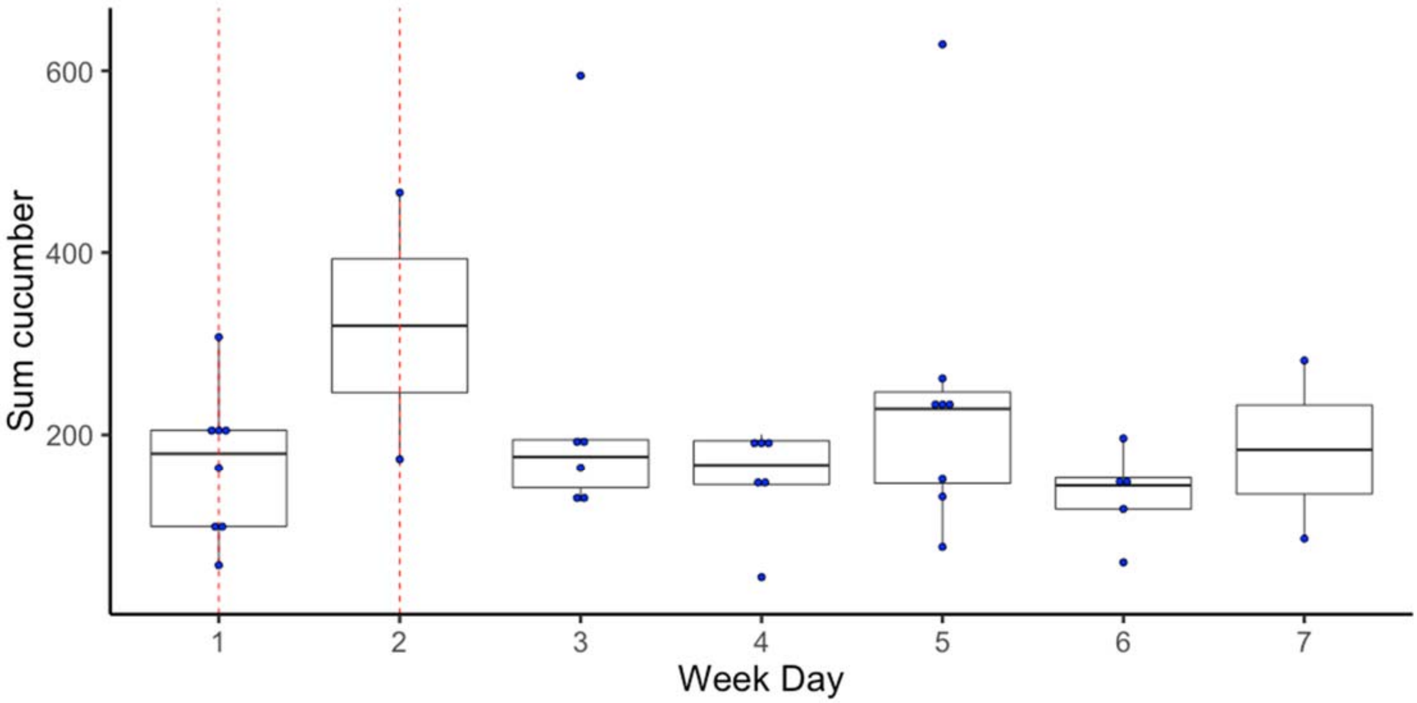

600
400
200
Sum cucumber
1
2
3
4
5
6
7
Week Day

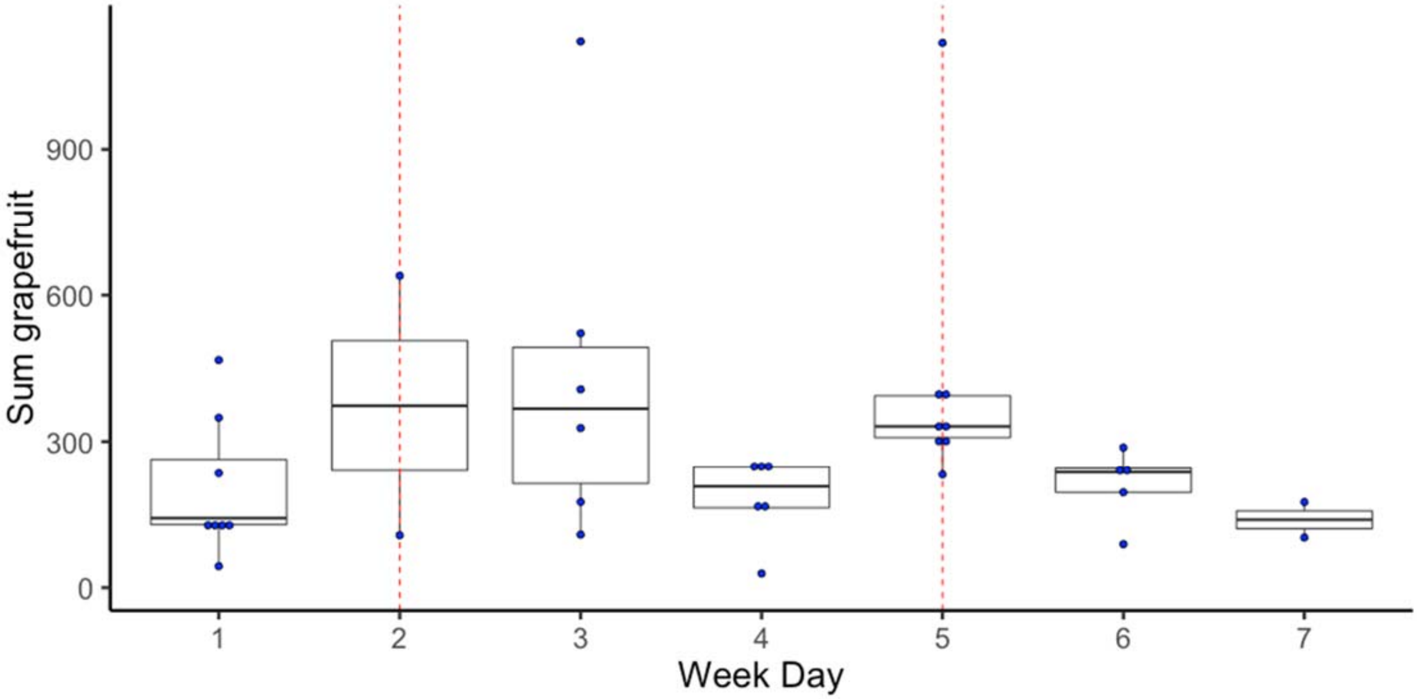

900
600
300
0
Sum grapefruit
1
2
3
4
5
6
7
Week Day

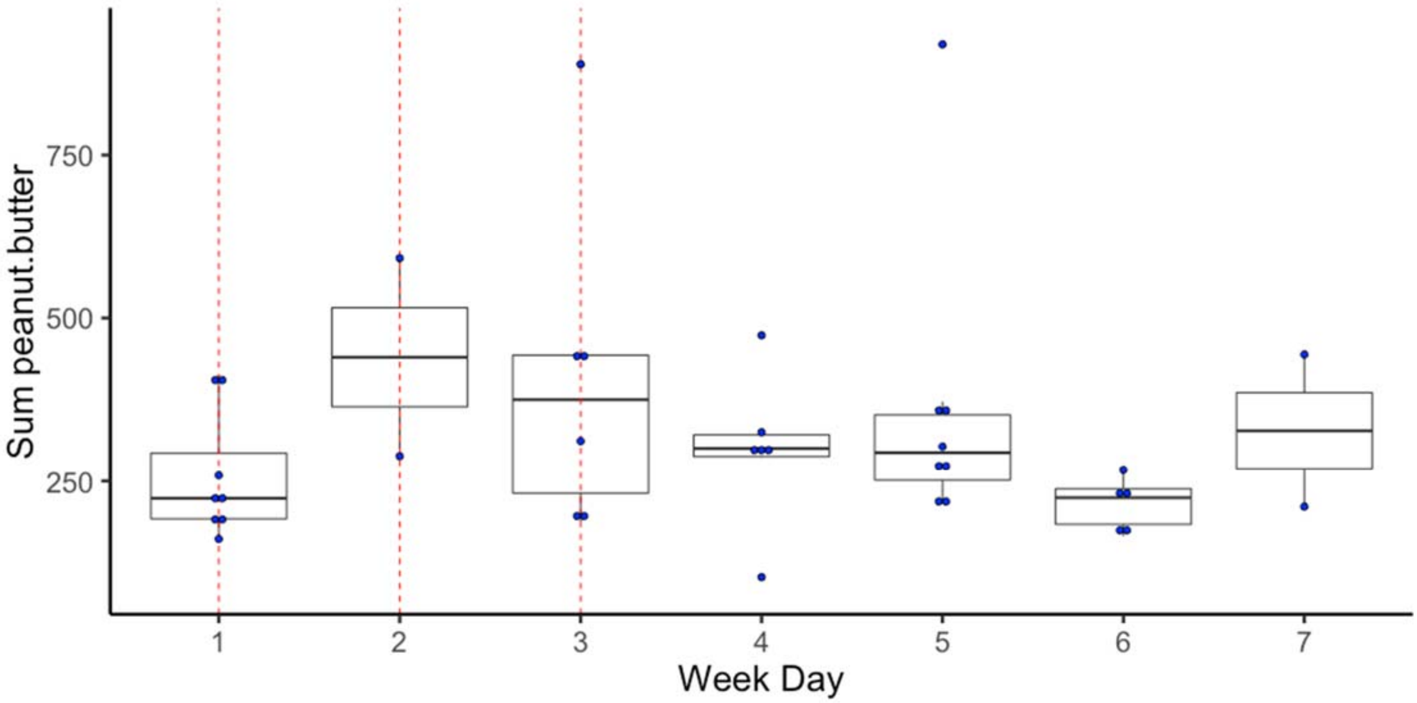

750
500
250
Sum peanut.butter
1
2
3
4
5
6
7
Week Day
