# Supplementary material for: Nutrimetabolomics reveals food-specific compounds in urine of adults consuming a DASH-style diet
Source: Sci Rep. 2020 Jan 24;10:1157. doi: 10.1038/s41598-020-57979-8 (PMC6981146; doi:10.1038/s41598-020-57979-8)
Supplement: Supplementary file 6 — Supporting Information 6. [file 41598_2020_57979_MOESM6_ESM.pdf]

**SUPPLEMENTAL METHODS**

# Nutrimetabolomics reveals food-specific compounds in urine of adults consuming a DASH-style diet

Nichole A. Reisdorph, Audrey E. Hendricks, Minghua Tang, Katrina A. Doenges, Richard M. Reisdorph, Brian C. Tooker, Kevin Quinn, Sarah J. Borengasser, Yasmeen Nkrumah-Elie, Daniel N. Frank, Wayne W. Campbell, and Nancy F. Krebs

## Controlled feeding study subjects

The current study utilized samples from a randomized, cross-over, controlled-feeding study that has been described previously.[1] A total of 333 volunteers contacted the study coordinator for participation in the study, 49 of them completed clinical screening, and 28 of the 49 screeners participated in the study. Details regarding study inclusion and exclusion criteria were included in the previously published study.[1] Nine of the 28 participants withdrew from the study due to time conflicts or inability to comply with study procedures for testing or dietary intervention. A total of 13 women and 6 men completed the controlled-feeding study [mean ± SEM age: 61 ± 2 y; BMI (in kg/m2): 31.2 ± 1.4]. At baseline, group mean blood pressures (BPs) were as follows: systolic blood pressure (SBP)/diastolic blood pressure (DBP): 130 ± 2/85 ± 2 mm Hg.[1] The study was conducted at Purdue University, West Lafayette, IN. Written informed consent was obtained from each subject before they entered the study. Subjects received monetary compensation after completion of the study. The Purdue University Biomedical Institutional Review Board approved the study procedures and documents. The study was registered at clinicaltrials.gov: NCT01696097.

## Experimental design

The 18-week study was chronologically comprised of a 2-wk pre-intervention (PI-1), the first 6-wk dietary intervention, a 4-wk dietary wash-out period that included a 2-wk pre-intervention (PI-2), and a second 6-wk dietary intervention (**Supplemental Figure S1**). Subjects were randomly assigned to consume either a DASH-style diet with lean pork (DASH-P) or chicken and fish (DASH-CF) as the predominant source of dietary protein during each of the two 6-wk interventions. Measurements were made during two weeks before and last two weeks of the two intervention periods (measurement periods).

**Diet intervention**

Subjects consumed their habitual diets during PI-1, wash-out, and PI-2 periods. After sample collection during pre-intervention, subjects were randomized to have a 6-week DASH-style diet (DASH-P or DASH-CF) with targeted percentages of energy derived from fat, carbohydrates, and protein of 27%, 55%, 18%.[2, 3] The controlled DASH-style diets include 55% of total protein intake from either lean pork or chicken and fish (tilapia fillets), with the remaining 45% of total protein intake from dairy, vegetable, and lean beef. Sodium intake was prescribed at an average level of ~2500 mg/d [3, 4] and adjusted with snacks and condiments as provided. **Supplemental Document S1_DASH Macros and Menus** includes an example of a DASH 7-day menu's macronutrient summary form. All meat products were provided to subjects while remaining food items listed on a detailed shopping list were purchased from a local grocery store. Both DASH-style diets (DASH-P and DASH-CF) targeted weight-maintenance.[5] Dietary adherence was maintained using a prescribed 7-d menu and a daily menu checklist. Dietary intakes at PI-1 and PI-2 periods were assessed by 3-d dietary records. Three-day dietary records completed during PI-1 and PI-2 and menu checklists from 3 days during the last 2 weeks of each intervention period were assessed for energy and nutrient contents by a registered dietitian with Nutrition Data

System for Research software (NDSR 2012; Nutrition Coordinating Center). Details regarding methods used to promote dietary compliance to achieve prescribed energy and macronutrients, as well as documentation procedures to measure their habitual dietary intake were published previously [1].

More information regarding participant recruitment and screening procedures, including inclusion criteria and a CONSORT flow diagram; dietary control and compliance techniques; and BP measurement and urine collection, processing and storage methods are published [1] and are also included in **Supplemental Figure S1**.

**Individual foods, diets and urine collections.**

Food listed on the seven daily menus (**Supplemental Document S1**) for the DASH-P and DASH-CF diets was purchased from a local grocery store for metabolomic analyses. Fresh, whole foods were randomly chosen for metabolomics analysis based on their presence in the dietary menus. Budgetary constraints limited the number of foods that could be analyzed using metabolomics. Because the focus of the study was on meat, all four types of meat included in the diet (pork, chicken, fish, and beef loin) were analyzed. Fruits and vegetables, if not pre-washed, were washed with tap water and prepared with inedible parts (i.e. leaves or peels) removed. Meat and eggs were cooked as instructed to match prescribed cooked weight. All samples (12 selected individual foods) were stored in a -80°C freezer and shipped with dry ice to the University of Colorado Anschutz Medical Campus, where lyophilization and methanol extraction were performed.

**Chemicals, standards and reagents**

Solvents used for compound extraction and LC/MS analysis were of HPLC or LC/MS-grade as follows: water and isopropyl alcohol from Honeywell Burdick & Jackson (Muskegon, MI); acetonitrile, methanol, chloroform, formic acid, and acetic acid and microcentrifuge tubes from Fisher Scientific (Fair Lawn, NJ); authentic standards from Avanti Polar Lipids Inc. (Alabaster, AL), Cambridge Isotope Laboratories (Tewksbury, MA) and Sigma Aldrich (St. Louis, MO); plastic pipette tips from USA Scientific Inc. (Ocala, FL); autosampler vials, glass inserts and caps from Agilent Technologies (Santa Clara, CA).

**Sample Preparation**

Individual food and urine samples were stored at -80 °C prior to sample preparation. Individual foods (including apple juice) were individually dried using a FreeZone 2.5 plus lyophilizer (Labconco, Kansas City, MO, USA). Dried samples were then divided and macerated using a chilled bio-pulverizer (BioSpec Products, Bartlesville, OK). Approximately 50 mg of each sample was measured into chilled microcentrifuge tubes and stored at -80° C prior to further processes and analysis. 10 µL of coffee was diluted with 90 µL of water, vortexed, aliquotted and stored at -80° C until analysis. An aliquot of coffee was also reserved for neat (i.e. unprocessed) analysis, and stored at -80° C until analysis.

Freeze-dried foods were removed from -80° C and allowed to thaw on ice. To each sample, 480 µl of chilled methanol, 10 µl each of a hydrophilic spike mix, hydrophilic positive control mix, hydrophobic spike mix and hydrophobic positive control mix were added (see Supplemental Table S1). Samples were gently vortexed for 10 seconds, placed at -80° C for 60 minutes, and then centrifuged 0° C at 18,000 x g for 15 minutes to facilitate protein precipitation. Supernatants were transferred to new microcentrifuge tubes and dried using vacuum centrifugation at 45° C for approximately 60 minutes. Each sample was suspended in 50 µl of 95:5 LC/MS grade water-

acetonitrile and gently vortexed for 30 seconds. Following a quick centrifugation, each sample was then divided into replicate 20 µl aliquots, while 10 µl was removed from each sample to generate a pooled QC sample. Samples were stored at -80° C until analysis.

Urine samples were analyzed neat (i.e. without any preparation) except that authentic standards were spiked into urine samples to monitor variability in instrument response or batch effects. A list of the authentic standards and their concentrations are presented in **Supplementary Table S1**.

**Liquid Chromatography Mass Spectrometry (LC/MS)**

Urine, individual food components, and composite daily diets were analyzed by reverse phase chromatography using an Zorbax Rapid Resolution HT (RRHT) SB-AQ, 1.8 micron (2.1 x 100 mm) analytical column and an Agilent Zorbax SB-AQ, 5 micron (2.1 x 12.5 mm) guard column (Agilent Technologies, Santa Clara, CA). An Agilent 1290 series high performance liquid chromatography (HPLC) pump was used (Agilent Technologies, Santa Clara, CA). An injection volume of 1 µL was used for all food and urine samples except for peanut butter which used a volume of 8 µL. HPLC flow rate was 0.250 mL/min with mobile phase A (water with 0.1% formic acid) and B (90:10 acetonitrile:water with 0.1% formic acid). The gradient was as follows for positive mode: 0-3.0 minutes 2% B, 3-10 minutes 2-60% B, 10-15 minutes 60-100% B, 15-20 minutes 100% B, 20-20.1 minutes 100-2% B, 20.1-30 minutes 2% B. Autosampler tray temperature was set to 4 °C and column temperature was set to 30 °C.

**Mass spectrometry (MS)**

The MS conditions for the urine, individual food and composite daily diet samples were as follows: Agilent 6520 Time-of-Flight (TOF-MS) with dual electrospray ionization (ESI) source, scan rate 2.21 spectra/second, mass range 50-1700 m/z, gas temperature 325 °C, gas flow 12.0

L/min, nebulizer 30 psi, skimmer 60 V, capillary voltage 4000 V, fragmentor 120 V, reference masses 121.050873 and 922.009798 (Reference mix, Agilent Technologies,).

**Quality control (QC)**

Total ion chromatograms (TIC) from the urine and food/composite diet sample and Instrument QC samples (injected after every 6 samples) were evaluated for retention time reproducibility using consistently detected compounds. The largest retention time variation was < 0.2 mins for urine and foods. Instrument QC sample were $\log_2$ transformed and analyzed to ensure that peak heights of endogenous compounds were reproducible. In the urine Instrument QC samples, 481 compounds were present in all samples, 476 of which had CVs <10%. In the food Instrument QC samples, 787 compounds were present in all samples, all of which had CVs <10%.

**Data processing**

Untargeted data mining (i.e. feature extraction) was performed using a so-called "recursive workflow", whereby data is extracted in two steps as follows. First, molecular features were extracted in MassHunter Profinder software (Agilent Technologies, Santa Clara, CA) using the Find by Molecular Feature (MFE) algorithm with the following parameters: spectral peak height filter 1,000 counts, chromatographic peak height 5,000 counts, charge states 1-2, allowed charge carriers proton, sodium, potassium, and ammonium, MFE quality score 80, retention time tolerance 0.30 minutes, and mass tolerance 15 ppm. Only compounds with two or more ions were retained. Data were imported into Mass Profiler Professional software (MPP, Agilent Technologies, Santa Clara, CA) and compounds present in preparation blanks and instrument blanks were removed. For the second step, compounds present only in foods or urine were exported as a composite .CEF file and this file was used as a target source for recursive feature extraction

in Profinder. For the recursive feature extraction, mass and time tolerances, and charge states, were the same as described above. Retention time was required for a match to MFE-extracted ion clusters. The chromatographic peak height was reduced to 4,000 counts.

All samples were processed in a single batch which enabled direct comparison of compounds found in different samples. The final data set was then re-imported into MPP for further processing, differential, and statistical analysis. For comparison of foods and urine, all data files were extracted in the same batch. To enable the separate analysis of only urine samples, data extraction was also performed on urine data files alone. This lessened the likelihood that extraction artifacts would be present in the final, urine-only dataset.

Following recursive feature extraction, the result is an ordered list of unnamed compounds labeled by their mass and retention time; a numerical value corresponding to the relative amount of that compound, a so-called peak area/volume or abundance, is also associated with each compound. To obtain a compound's abundance value, the mass spectral signal from all isotopes associated with a specific compound are merged across that compound's elution time. [6] Note that abundance values have no units, hence comparing the abundance of a compound between samples is considered relative quantitation, with results expressed as fold-change or fold-difference (i.e. differential analysis). Following feature extraction, abundance values were first converted from area to height; we have found abundance values are more reliable when this conversion is applied.[6] Due to the high variability in the initial starting material of all foods and the large degree of variance in matrix composition of foods, normalization was not used when foods and urine were combined in the same analysis. For the analysis of urine alone, data were normalized using total useful signal. To summarize, feature extraction is performed on each LCMS run as described. The abundance of all features extracted from a sample is summed and this is the Total Useful

Signal for that sample. This value is then used to normalize the abundance of each compound in the sample whereby the log2 abundance of the scaled total useful signal is subtracted from the log2 abundance of each compound in the sample.

**Compound annotation**

ID Browser within the Mass Profiler Professional (MPP) software v13.1 (Agilent Technologies, Santa Clara, CA) was used to putatively annotate compounds. This software utilizes an in-house database containing data from METabolite LINk (METLIN), Human Metabolome Database (HMDB), Kyoto Encyclopedia of Genes and Genomes (KEGG), Lipid Maps and an in-house authentic standard database. FooDB was searched separately. MPP uses isotope ratios, accurate mass, chemical formulas, and database scores (scale of 0 to 100) to annotate compounds by database ID, molecular formula, or compound number. The compound annotation corresponds a Metabolite Standards Initiative (MSI) level 3 identification.[7] A database score >50 out of a possible 100 was considered acceptable for annotation confidence; results were manually confirmed. Molecular formula generation included the following elements: C, H, N, O, S, and P. An error window of <10 ppm was used with a neutral mass range up to 2,000 Da. Database identifications were limited to the top 10 best matches based on score, and charge state was limited to a maximum of 2. A full list of food compounds is included as **Supplemental File "Uncurated Food Compound Lists".** Urinary compounds correlating with changes in blood pressure following DASH diet were subjected to tandem MS to improve confidence in identifications based on fragmentation information. Fragments were matched to reference standards from METLIN and NIST14 MS/MS spectral libraries.[8] All MS/MS-level, library-matched identifications are MSI level 2 based on the proposed minimum reporting by Sumner, et al.[9] Note that no MS/MS data

were matched to spectra from an in-house library comprised of data from authentic standards; in this case, identification would have been MSI level 1.

Annotated compounds were grouped into classes using the HMDB and Lipid Maps classification system. For the compound classes with four or fewer detected compounds in at least one of the two groups (urine, individual foods), that class was excluded for at least 2 of the following reasons: (1) most likely a false annotation, (2) below the detection level of the instrumentation, or (3) too many classes to display due to space limitations.

**Internet Search of FSCs**

Following annotation, a manual internet search was conducted to accomplish the following: 1) Determine the likelihood that an annotation was correct, 2) Determine if a compounds could be classified as a FSC, 3) Determine if FSCs had previously been found in a specific food, and 4) Determine if FSCs had previously been found in any food. Compounds that were not annotated or not likely to be accurate annotations were omitted from further analysis. Results are shown in **Supplemental File- FSC**. This is a somewhat subjective exercise, however, certain rules were applied as described below. Because the goal of the study was not to systematically validate biomarkers of food intake, but rather to provide proof-of-principle for an overall strategy for discovering new biomarkers of food intake, the recently published guidelines for evaluating biomarkers were not followed.[10] Rather, internet searches were conducted by first searching the compound against the HMDB using HMDB ID, if available. When a compound was present in HMDB, an external link to FooDB was also searched if a FooDB ID was present. Where available, the section on “Associated Foods” was searched to determine if the food of interest was listed there. If no HMDB ID was available, compounds were searched against KEGG using the KEGG ID or LipidMaps using the LMP ID. If no or limited

information was available for a compound, compound names were pasted into the web-tool Knapsack and a Google search was conducted.

Following initial searches, compounds and foods were searched as together using Google, PubMed, and Google Scholar. For example, one of the metabolites found in coffee was from the herb Rue. A Google search of "rue + coffee" revealed that, in Ethiopia, rue is brewed with coffee. A summary of the results, including links or publication IDs, were placed into the "Notes" column of **Supplemental File- FSC**.

Although this was a somewhat subjective exercise, rules and guidelines were adhered to as follows:

Compounds were marked as "previously determined to be in that food" if a search revealed that a compound had been previously found in that food. For example, HMDB listed 4-hydroxydiphenylamine as "found in pomes"; since pomes are apples, this compound was marked as having been previously determined to be in apples. Compounds were marked as "Probably/Possibly in the food" if it was found in that food or similar foods; for example, a FSC detected in apple in the current study was considered probably/possibly present in apple if it had been found in any fruit. A FSC detected in blueberry was considered probably/possibly present if it had been found in any type of berry. Compounds that had been found in any food, food-product, or plant were marked as "Found in Some/Any other Food". Compounds such as additives, flavorings, toxins, and medications that showed evidence of being used in relation to the food of interest, were included as "Found in Some/Any other Food" and marked as "Exogenous". Given the ubiquitous nature of most lipids, lipid species were considered to be "Found in Some/Any other Food" unless there was evidence to the contrary. Bile acids, microbiome-related metabolites, peptides, and other compounds that were found in KEGG

pathways, most of which were detected in meat samples, were treated similarly. In general, "Exogenous" compounds were those that could be considered exogenous to plants, animals, or humans.

### Statistical Methods

All statistical analyses were completed in R Studio v.1.1.456. Linear mixed effects models were run using the function lme from the nlme package.

### Relative Metabolism of Individual Foods

For the seven foods consumed consistently in both DASH-style diet periods (apple juice, beef tenderloin, blueberries, broccoli, cucumber, grapefruit, and peanut butter), we summed the abundances of FSCs that were also found in urine (**Table 1** column 4) to derive a food-specific signature. For example, the abundances of all 64 blueberry FSCs were used to generate a blueberry-specific signature. Other meats and apples were excluded from the relative metabolism analysis as these foods were either excluded entirely or partially from one of DASH-style diet periods, limiting the sample size. For each DASH-style diet intervention period, urine samples were collected in the pre-diet period and the post-diet period. A rigid schedule was kept for food consumption, with the same foods being consumed on each diet week day; for example, grapefruit was always consumed on Diet Days 2 and 5. However, the timing of urine collection varied by participant. For example, as shown in Supplemental Figure S3, during Study Week 6, on day 29, urine was collected from 4 participants (the number in parentheses). On day 30, urine was collected from 1 participant while on day 36, urine was collected from 4 participants. Therefore, on Diet Week Day 1, a total of 8 urine samples were collected. Because urines were collected at various Diet Week Days, there was a variable amount of time between urine collection and consumption of a particular food. For example, if urine was collected on Diet Day

1, it had been 3 days since the participants had consumed grapefruit. Using this information, the number of days since last consumption was determined for each food. To determine relative metabolism of foods, a linear mixed effects model was used with individual participant as the random effect, the food specific signature as the outcome, and number of days between when a specific food was consumed and when the urine collection occurred as the predictor. Significance was assessed at a Bonferroni correction for the seven foods ($\alpha = 0.05 / 7 = 0.00714$). Models also were run removing outliers and adjusting for batch to ensure consistency.

**Correlation of urinary compounds with blood pressure**

We used two approaches to determine if associations exist between urinary compounds and BP levels in these participants. For all analyses, only compounds present in at least 50% of the urine samples were included. Zero values were assumed to be missing and were removed from statistical models for that compound. We separately considered both urinary FSCs and all other compounds found in urine; the source of these other compounds was unknown but could be classified as endogenous, food-related, or from other exogenous sources (eg. Environmental contaminants).

The first approach assessed if the relative abundance of each FSC or other compound in urine was associated with SBP or DBP. Data from all 76 measurements (19 subjects, each with four measurement periods) were included. We used a linear mixed effects model with a random intercept for individual participants and BP level as the outcome. The relative amount of a FSC or other compound in urine, participant body mass index, age, sex, and batch were the predictors. A Bonferroni corrected threshold adjusting for the number of tests (i.e. ($\alpha = 0.05 / (90 * 4) = 0.00014$) was used to assess significance. Nominal significance ($p < 0.05$) was also reported for

exploratory purposes. Seventeen nominally significant ($p < 0.05$) compounds were manually re-extracted from the raw data, resulting in 16 compounds with non-zero quantities. This was accomplished using extracted ion chromatograms (EICs or XICs) generated in MassHunter software. The analysis was repeated on these updated values both including and excluding the samples with compound values of zero.

The second approach was to assess if the relative abundance of each FSC or other compound in urine while the participants consumed the DASH-style diet was associated with changes over time in systolic or diastolic BP during the controlled feeding periods (end of intervention BP minus pre-intervention BP). Data from 38 urine samples and 38 BP change values were included. We used a linear mixed effects model with a random intercept for individual participant, change in systolic or diastolic BP as the outcome, and compound abundance in urine collected while the participants consumed the DASH-style diets and batch as predictors.

[1] Sayer, R. D., Wright, A. J., Chen, N., Campbell, W. W., Dietary Approaches to Stop Hypertension diet retains effectiveness to reduce blood pressure when lean pork is substituted for chicken and fish as the predominant source of protein. *Am J Clin Nutr* 2015, *102*, 302-308.
[2] Karanja, N. M., Obarzanek, E., Lin, P. H., McCullough, M. L., *et al.*, Descriptive characteristics of the dietary patterns used in the Dietary Approaches to Stop Hypertension Trial. DASH Collaborative Research Group. *Journal of the American Dietetic Association* 1999, *99*, S19-27.
[3] Svetkey, L. P., Sacks, F. M., Obarzanek, E., Vollmer, W. M., *et al.*, The DASH Diet, Sodium Intake and Blood Pressure Trial (DASH-sodium): rationale and design. DASH-Sodium Collaborative Research Group. *Journal of the American Dietetic Association* 1999, *99*, S96-104.
[4] Sacks, F. M., Svetkey, L. P., Vollmer, W. M., Appel, L. J., *et al.*, Effects on blood pressure of reduced dietary sodium and the Dietary Approaches to Stop Hypertension (DASH) diet. DASH-Sodium Collaborative Research Group. *The New England journal of medicine* 2001, *344*, 3-10.
[5] Medicine, I. o., *Dietary Reference Intakes for Energy, Carbohydrate, Fiber, Fat, Fatty Acids, Cholesterol, Protein, and Amino Acids*, The National Academies Press, Washington, DC 2005.
[6] Reisdorph, N. A., Cruickshank-Quinn, C., Nkrumah-Elie, Y., Reisdorph, R., Application of Metabolomics in Lung Research. *Methods Mol Biol* 2018, *1809*, 263-288.

[7] Sumner, L. W., Amberg, A., Barrett, D., Beale, M. H., *et al.*, Proposed minimum reporting standards for chemical analysis Chemical Analysis Working Group (CAWG) Metabolomics Standards Initiative (MSI). *Metabolomics* 2007, *3*, 211-221.
[8] Yang, X., Neta, P., Stein, S. E., Quality Control for Building Libraries from Electrospray Ionization Tandem Mass Spectra. *Analytical Chemistry* 2014, *86*, 6393–6400.
[9] Sumner, L. W., Amberg, A., Barrett, D., Beger, R., *et al.*, Metabolomics Society 2007.
[10] Pratico, G., Gao, Q., Scalbert, A., Vergeres, G., *et al.*, Guidelines for Biomarker of Food Intake Reviews (BFIRev): how to conduct an extensive literature search for biomarker of food intake discovery. *Genes Nutr* 2018, *13*, 3.
